# Electrocatalysis with Molecular Transition-Metal Complexes for Reductive Organic Synthesis

**DOI:** 10.1021/jacsau.2c00031

**Published:** 2022-05-31

**Authors:** Nicolas Kaeffer, Walter Leitner

**Affiliations:** Max Planck Institute for Chemical Energy Conversion, Stiftstrasse 34-36, 45470 Mülheim an der Ruhr, Germany

**Keywords:** Molecular electrocatalysis, Organic synthesis, Reduction reactions, Organometallic catalysis, Sustainable resources

## Abstract

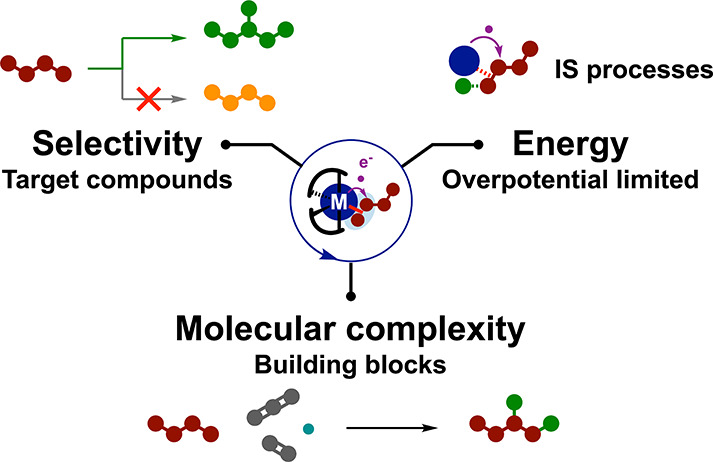

Electrocatalysis enables the formation or cleavage of chemical bonds by a genuine use of electrons or holes from an electrical energy input. As such, electrocatalysis offers resource-economical alternative pathways that bypass sacrificial, waste-generating reagents often required in classical thermal redox reactions. In this Perspective, we showcase the exploitation of molecular electrocatalysts for electrosynthesis, in particular for reductive conversion of organic substrates. Selected case studies illustrate that efficient molecular electrocatalysts not only are appropriate redox shuttles but also embrace the features of organometallic catalysis to facilitate and control chemical steps. From these examples, guidelines are proposed for the design of molecular electrocatalysts suited to the reduction of organic substrates. We finally expose opportunities brought by catalyzed electrosynthesis to functionalize organic backbones, namely using sustainable building blocks.

## I Introduction

**Figure 1:**
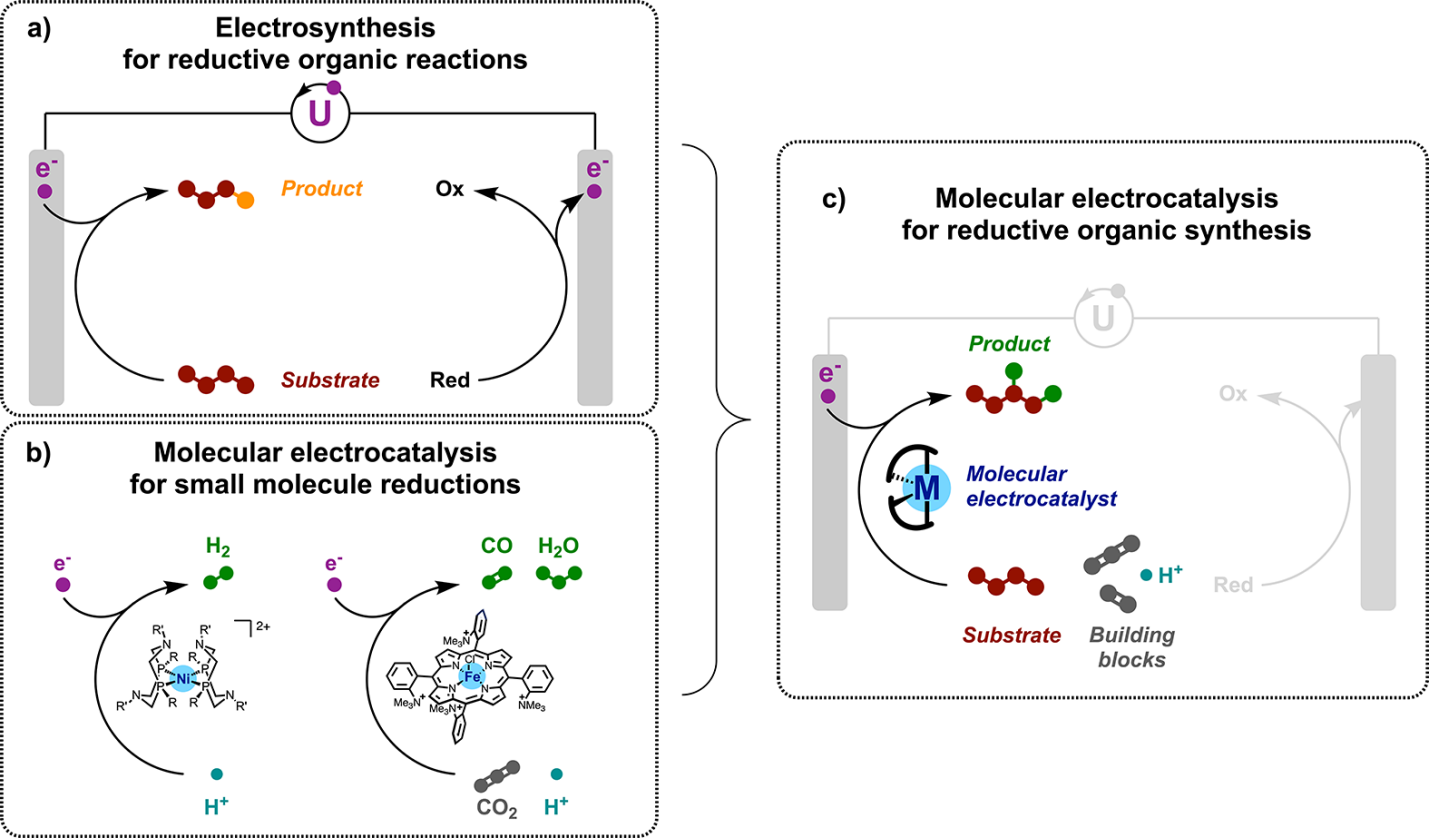
(a) Electrosynthesis for reductive organic reactions, (b) molecular electrocatalysis for small molecule reductions, and (c) the merger of both in molecular electrocatalysis for reductive organic synthesis.

Renewable electricity offers a unique, sustainable opportunity to make or break chemical bonds and hence to produce chemical compounds. This so-called electrosynthetic approach^1−8^ (Figure 1a) bypasses the need for sacrificial, waste-generating reagents used in thermal redox conversions ubiquitous in organic synthesis. In the thermal approach, key operational factors as selectivity and energy efficiency are highly being improved via the development of tailored catalysts, among which molecular organometallic species are prominent at a high functionalization level.^9−11^ In comparison, the number of molecular catalytic systems purposely designed for the electrosynthesis of complex chemicals remains moderate, especially in the frame of reductive transformations.^7,12−14^ The limited exploration of such catalysts—*electrocatalysts*—for organic synthesis is even more striking when compared to the vibrant field of molecular electrocatalysis for small molecule (H^+^, CO_2_, N_2_) conversions^15−21^ (Figure 1b).

In this Perspective, we aim to show the potential that molecular electrocatalysts can bring into electrosynthesis (Figure 1c). We showcase the possible impact for the specific case of reduction reactions adding value to organic chemicals.

First, introductory remarks discuss the opportunities brought by electrocatalysis in general and molecular electrocatalysis in particular to address challenges in electrosynthetic conversions. These points are then illustrated with selected case studies on reductive syntheses of organic compounds successfully fostered by molecular electrocatalysts: C=O hydrogenation, 1,2-dehalogenation, aryl–aryl coupling, and unsaturated C–C bond hydrocarboxylation. In a back and forth between mechanistic considerations and reactivity, we rationalize how molecular electrocatalysis was effective in tuning the activity, selectivity, energy efficiency, and scope of the electrosyntheses under consideration. The case studies discussed here show that efficient molecular electrocatalysts not only act as effective inner-sphere redox shuttles but also combine the features of organometallic catalysis^22^ by facilitating chemical steps. Joining these manifolds, molecular electrocatalysis opens a promising perspective in addressing the energy efficiency and resource efficiency via reactivity and selectivity control in the reductive upgrading of organics.

Taking lessons from these established examples, we open our discussion by proposing guidelines for the design of molecular electrocatalysts suited to the reductive electrosynthesis, in the aim to widen the scope of building blocks and substrates. We then expose possible opportunities that catalyzed electrosynthesis brings in organic reductions (hydrofunctionalization, C–C couplings) and particularly toward the substitution of waste-generating reagents for sustainable resources (H_2_O, O_2_, CO_2_, N_*x*_O_*y*_) in the functionalization of added-value chemicals. We conclude with a zoom out to comprehend the counter oxidation in the overall reaction and implementation into devices.

## II General Remarks

### II.A Energy and Chemical Considerations in Electrosynthesis

Among the tremendous number of organic electrosyntheses developed to date,^1−6,23−25^ the operation in two-electrode electrochemical cells is common, with the use of intensity-potential power generators to supply the electron/hole redox equivalents to the reaction.^26^ Such setups are readily at hand and straightforward to use. In these setups, the difference of potentials between the working electrode (*E*_WE_) and the counter electrode (*E*_CE_) is known as the cell voltage *U*_app_ = *E*_WE_ – *E*_CE_ and relates the respective electronic energies. As the focus of this Perspective is reduction reactions, we consider here the working electrode as the cathode and the counter electrode as the anode. *E*_WE_ thus gives the energy of the electrons injected into the reductive electrosynthetic transformation. This value is of primary importance as a direct measure of the *driving force* provided for the reaction of interest. The flow of electrons passing through the cell is indicated by the cell current *I*_app_, which thus sizes the *rate* of delivery of these redox equivalents. The two-electrode electrosynthetic setups are principally operated under a fixed applied voltage *U*_app_ (*potentiostatic* conditions) or under a fixed applied current *I*_app_ through the cell (*galvanostatic* or *amperostatic* conditions). However, both approaches lack a precise control of *E*_WE_ on an absolute scale. This point is circumvented in three-electrode setups fitted with an additional reference electrode of known and constant absolute potential *E*_RE_. Such a configuration permits setting *E*_WE_ against a reference potential and thus accesses a control of *E*_WE_ on an absolute scale and thereby over the driving force applied to the reaction. Yet, three-electrode configurations require more specific potentiostats, whose cost and operation may be prohibitive. Innovations on the market to offer modular potentiostats equipped with high power amplifiers needed for electrosynthetic processes, analytical options, and user-friendly interfaces at affordable costs are thus highly sought for.^27^

**Figure 2:**
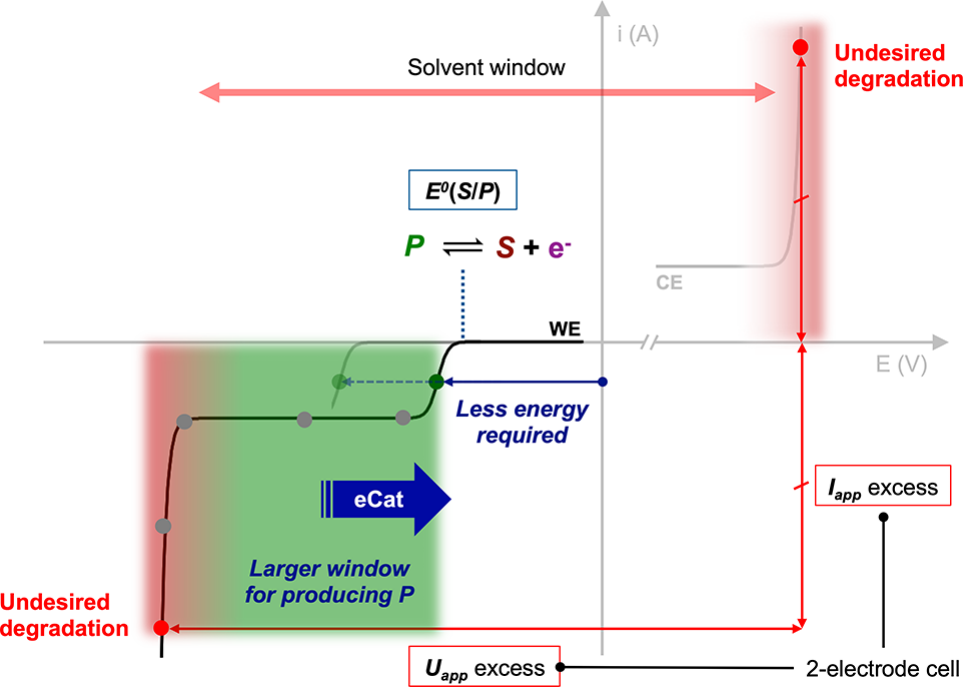
Electrosynthetic reductions: challenges in two-electrode cell configuration and advantages brought by electrocatalysis. WE, working electrode; CE, counter electrode.

Concerning chemical aspects, the fates of many electrosynthetic transformations in terms of conversion and selectivity are highly dependent on *E*_WE_, as will be exemplified in the case studies below. For instance, a too-moderate applied potential does not provide a sufficient driving force for the electrocatalytic reaction of interest to proceed, while a too-large applied potential may trigger competitive undesired side reactions, for instance by over-reducing intermediates or products, and/or by degradation of the media (Figure 2). The latter case is most probably encountered in the many two-electrode electrosynthetic systems operated at excessive cell voltage (typically *U*_app_ > 10 V) or current density (typically *I*_app_ > 10 mA/cm^2^) on bare, simple metallic or carbonaceous electrodes. We note that the undesired reactivity at high applied voltage is of concern not only for substrates/products but also for solvents, supporting electrolytic salts, and electrodes^28^ used to operate the cell. Indeed, the electrochemical stability window of common electrolytes does not span over more than 5 V^29^ and is thus surpassed either in the reductive region or in the oxidative region at strong cell voltage, causing degradation of the solvent upon operation.

In addition, in this Perspective we debate electroreductions that are by definition half-reactions and balanced in practice by a counterpart oxidative half-reaction. The anodic half-reaction engages the same amount of oxidizing equivalents (holes) as electrons injected in the reduction, and the second half-reaction should be taken into account when the chemical balance of the overall reaction is considered.

Concerning energy aspects, a large cell voltage can lead to a potential at the working electrode exceedingly more negative than the standard potential *E*^0^(***S***/***P***) of the redox couple for the interconversion of substrate ***S*** with product ***P***, as measured by the *overpotential* η = |*E*_WE_ – *E*^0^(***S***/***P***)|.^30,31^ Large overpotentials reflect important (electronic) energy losses and thus hinder the energy efficiency of the system. Having control of *E*_WE_ on an absolute scale, the most instrumental way to minimize the excess driving force for the targeted electrosynthetic reaction is the use of an electrocatalyst (Figure 2).

### II.B Role of the Electrocatalyst

**Figure 3:**
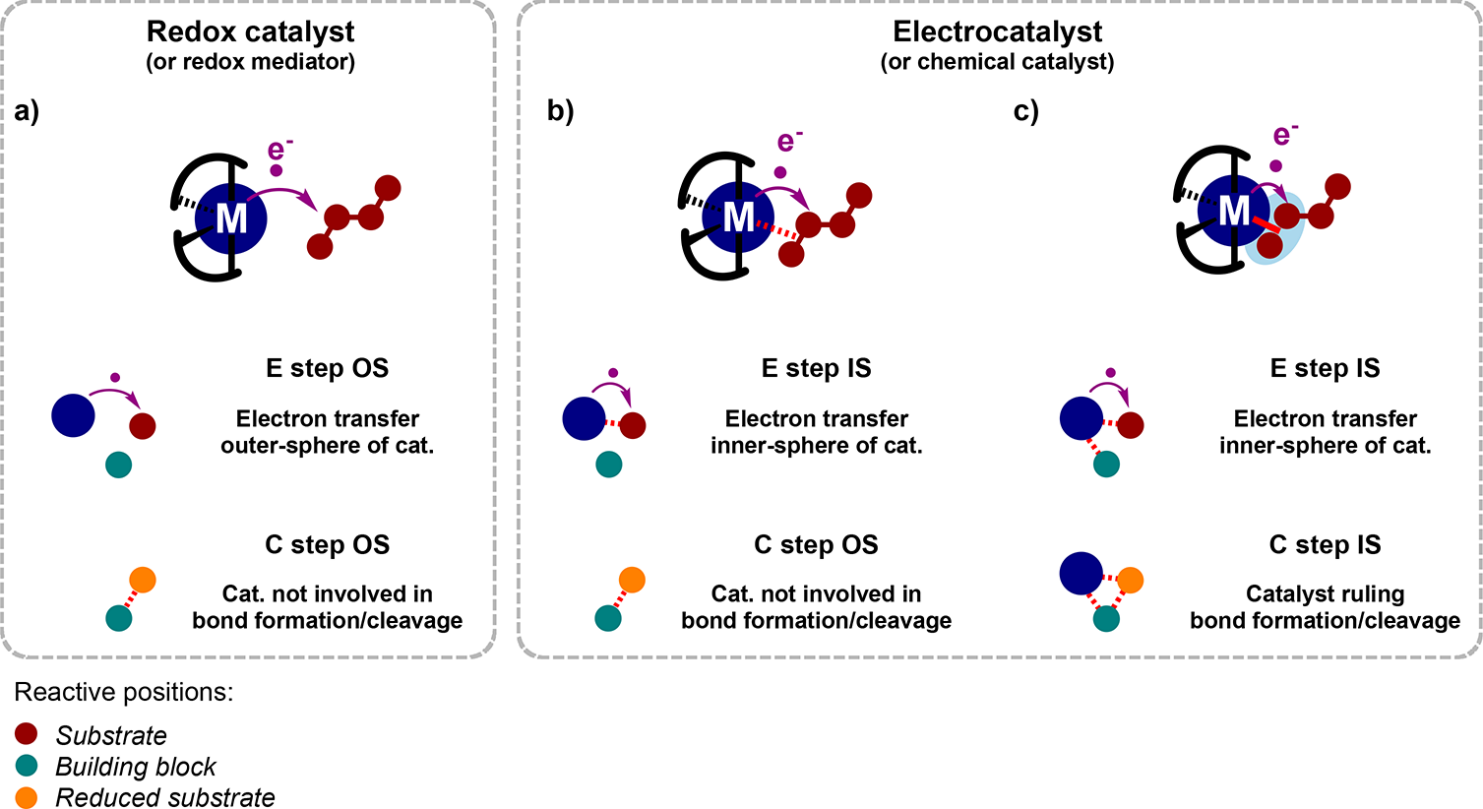
Redox catalyst (a) versus electrocatalyst (b, c): schematic representation of electron transfer modes and involvement in the electrochemical (E) and chemical (C) steps. Cat., catalyst; IS, inner sphere; OS, outer sphere.

In a molecular electrocatalytic cycle that comprises a sequence of electron transfer (E) and chemical (C) steps, the diffusing species that shuttles the electrons from the electrode surface to the substrate(s) can act following different modes. The precise action mode has a dramatic influence on the outcome of the electrosynthetic reaction. This differentiation is yet not always precisely specified in the literature, reflecting terminologies such as *mediator*, *redox catalyst*, and *electrocatalyst* being often abusively assimilated. In their general definition, a *mediator* or a *catalyst* can transfer electrons to the substrate following two different modes: in an *outer-sphere* (OS) or an *inner-sphere* (IS) fashion,^32,33^ whose contrast is established by distinguishing *redox catalysis* from *chemical catalysis* (Figure 3).^31,34^

In redox catalysis, the electron transfer takes place in the absence of appreciable electronic interactions between the catalyst and the substrate and can be described within the Marcus–Hush–Levich (MHL) model characterizing outer-sphere electron transfers^31^ (a similar expression of the electron transfer rate constant has been recently reported for IS processes, although at an electrode surface^35^). In the case of OS electron transfer, the redox catalyst (or redox mediator) is thus merely a shuttle for charges from the electrode surface to the substrate, intervening in E steps of the electrocatalytic cycle (Figure 3a). Notably, a redox catalyst is not involved in activation of the substrate via an adduct and does not modify the transition states of the C steps in the electrocatalytic cycle.

In contrast, an electron transfer occurring as an inner-sphere process (chemical catalysis) is correlated with a bonding interaction between the catalyst and the substrate.^31^ This action mode is the one followed by an electronically activated chemical catalyst and escapes the MHL model for OS electron transfer. In this Perspective, we refer to the term *electrocatalyst* for molecular homogeneous catalysts proceeding via IS electron transfers, by extension of the historical definition at electrode surface.

Further distinctions can be drawn within inner-sphere electron transfers. If bonding exists at the transition state of electron transfer only, the electrocatalyst facilitates the electron transfer E steps but not the C steps (Figure 3b). On the other hand, the contact may be more intimate and involve the formation of a substrate–catalyst adduct—an intermediate—for instance, by coordination of the substrate to a transition metal complex catalyst, as commonly encountered in catalytic transformations of organics. Here, the electrocatalyst not only eases the electron transfers (E steps) but is also brought to play a central role in the activation of the substrate and the elementary bond formation processes, ruling the C steps (Figure 3c).

Whether the catalyst is a redox catalyst or an electrocatalyst is of paramount importance. Indeed, only an electrocatalyst, by virtue of intimate contact and activation of the substrate, can substantially lower the activation energy of the kinetically rate-determining step that is responsible for the so-called electrochemical *overpotential requirement*.^34^ By facilitating the kinetics of the electrochemical transformation, an electrocatalyst limits the energy losses to reach substantial turnover and conversion. In addition, electrocatalysts may also promote the concertedness of electron transfers with bond formation or scission. This feature has major implications regarding selectivity, as will be developed in the cases studied below.

Finally, we want to note that electrochemically assisted catalysis, which relies on an electrochemical activation of the catalyst, also opens up a great deal of reactivity for redox neutral transformations.^36−38^ We however do not discuss the approach in detail in this Perspective since our focus is set on reductive synthesis, thus with a net consumption of electrons in the half-reaction of interest.

### II.C Performance Metrics for Electrocatalyzed Synthesis

To address the reach of electrocatalysis in the frame of organic transformations, a sound evaluation of the performances of electrocatalysts and the derivation of structure–activity relationships are essential.

**Figure 4:**
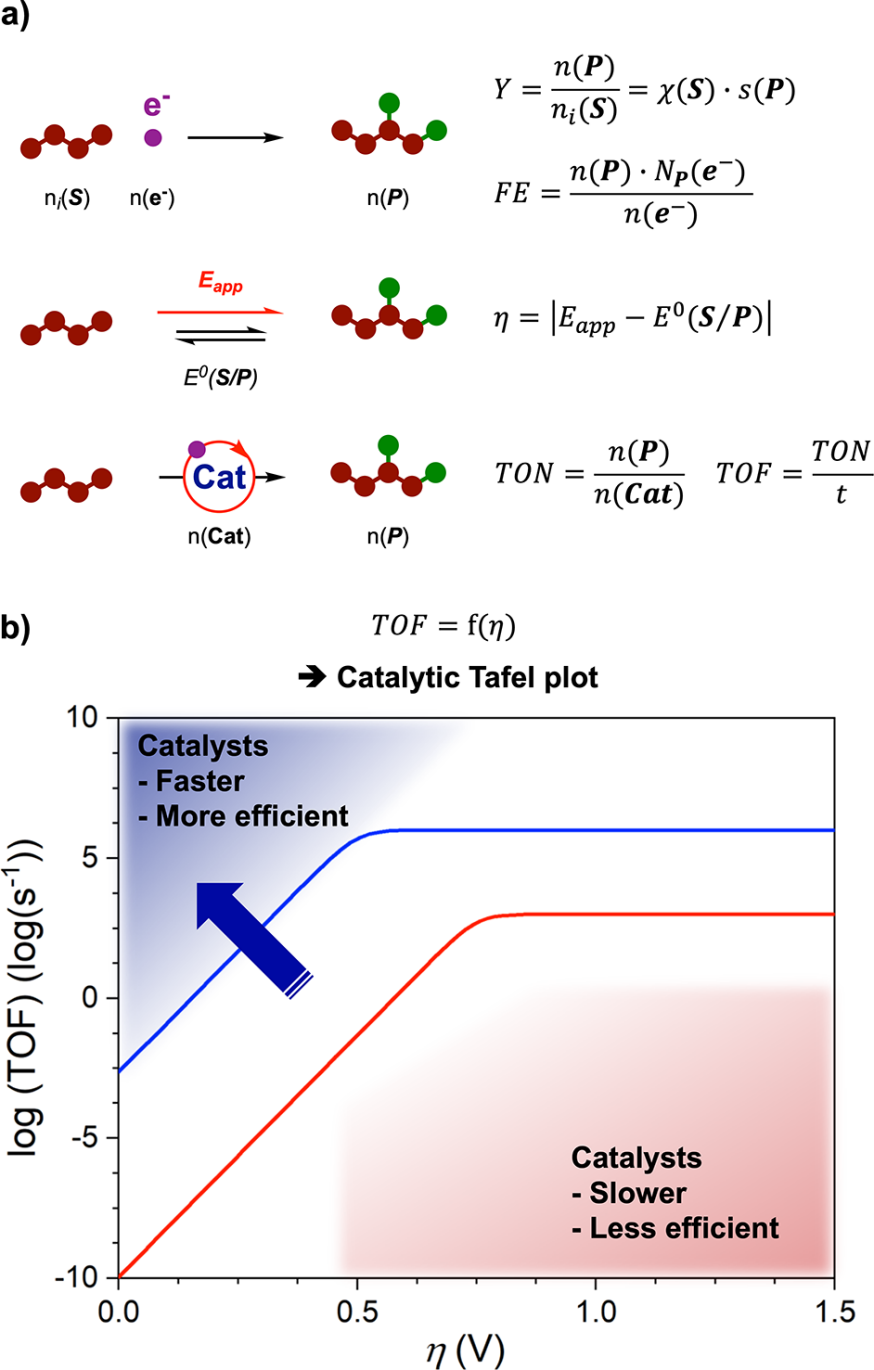
(a) Metrics to evaluate molecularly electrocatalyzed reductive synthesis and (b) illustrative catalytic Tafel plot. *Y*, yield; χ, conversion; *s*, selectivity; FE, Faradaic efficiency; *E*^0^(***S***/***P***), standard potential of the ***S***/***P*** redox couple; η, applied overpotential; TON, turnover number; TOF, turnover frequency; *n*(***A***), moles of species ***A***; *N*_P_(e^–^), electron stoichiometry to reduce ***S*** in ***P***; *t*, time.

Regarding activity, evaluating electrocatalyzed organic synthesis requires a combined analysis of chemicals and energy conversions (Figure 4a). To assess the transformation of chemicals, metrics utilized in classical chemical synthesis, such as substrate conversion, selectivity, and product yield, remain of course relevant.

Concerning energy conversion, an important figure of merit resides in the Faradaic efficiency (FE) or Faradaic yield, which measures the fraction of charges injected in the electrosynthetic cell that are actually directed to the redox transformation of interest. A Faradaic efficiency of unity indicates that all the injected charges are used for the desired substrate conversion. At variance, low FEs imply that charges are wasted in redox side reactions, which translates into a poor (electronic) energy efficiency of the electrosynthetic system under consideration. A careful evaluation of the FE is thus important, even if the redox equivalent (electrons/holes) obtained from electricity may be considered as a cheap resource compared to the engaged organic backbones. The energy brought to the electrocatalyst can also be interrelated with the substrate conversion efficiency by expressing the observed catalytic rate or turnover frequency (TOF) as a function of excess driving force, i.e., applied overpotential η. Such η–TOF relations introduced by Savéant and co-workers as *catalytic Tafel plots*(39,40) are a powerful tool to compare the efficiencies of molecular electrocatalysts for a given electrosynthetic conversion (Figure 4b). This analysis has been to date mostly exploited in the transformation of small molecules for energy conversion^31,41,42^ (H^+^, CO_2_, O_2_) but can easily be extended to the conversion of organics.^12^

In addition to the reaction metrics, analytical characterizations coupled to electrocatalysis have seen major progress during the past three decades. A large portofolio of spectroscopic or analytical techniques (e.g., UV–visible, IR, resonance Raman, EPR, NMR, XAS, TEM, MS) for *in situ* or *operando* characterization is now available to access in-depth molecular-level mechanistic information.^43,44^

## III Case Studies

Homogeneous electrocatalysis applied to the conversion of organic compounds is often based on catalytic entities prepared by introduction of a metallic salt and a ligand within the electrolysis cell.^3,45^ This approach avoids the prior isolation of the molecular (pre)catalyst and certainly offers a practical, highly versatile way to screen potential electrocatalytic species. However, the nature of the actual electrocatalytic entity produced *in situ* remains uncertain. This approach is thus ideally complemented by engaging the corresponding molecularly defined species to validate the identity of the active center and relate structure to activity. In this Perspective, we restrain our discussion to well-defined molecular electrocatalysts for a more precise discussion of the interplay between catalytic mechanism and operational factors such as substrate scope, activity, and selectivity.

We first (section III.A) illustrate that applying well-controlled potentials to precisely access the low-valent active form(s) of the electrocatalyst is an effective strategy to trigger the desired reactivity (e.g., hydride transfer, C–C coupling) while avoiding side reactions. In the following section (section III.B), we compare redox catalysts acting in an OS manner and electrocatalysts operating in an IS fashion and show that the latter provide definite advantages in terms of selectivity and energy efficiency. We finally (section III.C) highlight that electrocatalysts displaying features remanent of organometallic catalysis afford upgrading molecular complexity from simple building blocks.

### III.A Controlling the Electrochemical Potential

#### III.A.1 Hydrogenation of Ketones: Outcompeting Hydrogen Evolution

**Figure 5:**
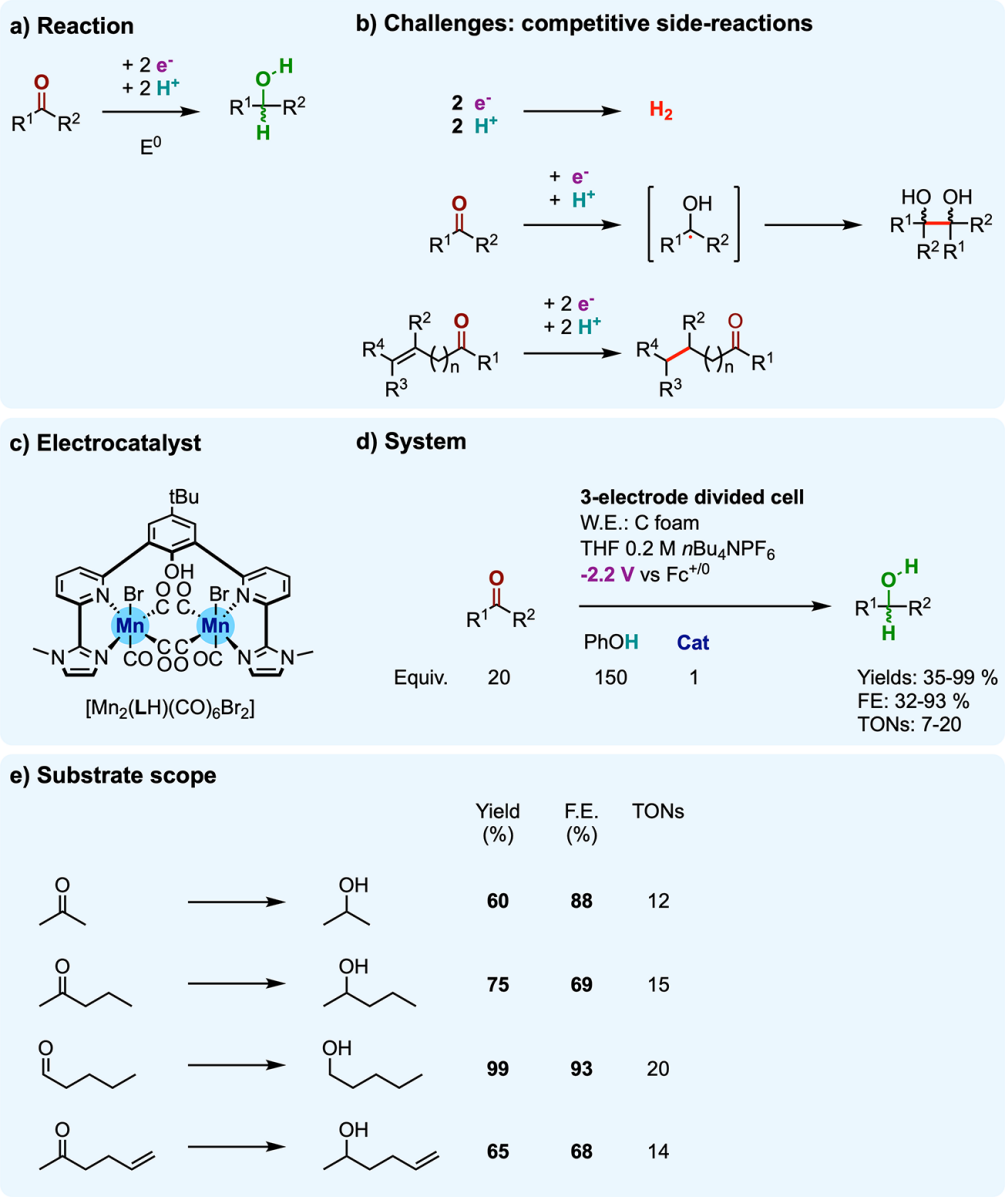
Electrocatalytic hydrogenation of unsaturated C=O bonds in ketones and aldehydes: (a) reaction; (b) challenges; (c) molecular electrocatalysis with [Mn_2_(**L**H)(CO)_6_Br_2_] under (d) the operating conditions applied to (e) substrate scope.

The hydrogenation of organic ketones and aldehydes to alcohols is an important part of the organic chemistry toolbox, with the electrochemical version (Figure 5a) finding interest in the synthesis of fuels and chemicals via biomass derivation.^46^ A first challenge regards selectivity and is transversal to all electrocatalytic hydrogenations: hydrogen evolution stands as a major competitive process. In particular, as the standard potentials for carbonyl/alcohol couples are generally close to that of H^+^/H_2_ (e.g., *E*^0^(acetone/isopropanol) = 0.12 V vs RHE, from ref (47)), hydrogenations of carbonyl groups do not possess a substantial thermodynamic advantage over hydrogen evolution. Another important challenge is here to reduce the carbonyl to the alcohol in a chemoselective way, avoiding the ketyl radical coupling and the hydrogenation of other unsaturations (e.g., C=C) when present (Figure 5b).^48,49^ To drive carbonyl hydrogenation to the desired selectivity, molecular electrocatalysts have been investigated,^50,51^ mostly based on Ni,^52,53^ Fe,^52,53^ Ru,^54^ and Rh^55−57^ metal complexes, with a body of work using homogeneous and immobilized Rh bipyridine type complexes.^55−57^

Recently, Siewert and Fokin disclosed the electrochemical hydrogenation of ketones and aldehydes using a well-defined molecular catalyst based on earth-abundant manganese, namely the binuclear Mn complex [Mn_2_(**L**H)(CO)_6_Br_2_] (Figure 5c).^58^ This complex had been previously established as an electrocatalyst for CO_2_ reduction.^59^ With the use of phenol as a proton source, a range of aliphatic ketones and aldehydes were electrocatalytically converted to their alcohols (Figure 5d,e), within good to excellent yields (60–99%; 12–20 TONs). These electrocatalyzed carbonyl hydrogenations are achieved at a moderately low applied potential (−2.2 V vs Fc^+/0^) translating in the case of acetone-to-isopropanol reduction to low overpotential (roughly 160 mV). At contrast, under the same applied potential, the reduction of acetone does not occur in the absence of the catalyst, in line with an apparent overpotential requirement at least 1 V higher at the bare electrode. This point further highlights how, by affording electrochemical carbonyl hydrogenation close to thermodynamics, the electrocatalyst provides a clear advantage in terms of energy efficiency. Furthermore, the electrocatalyzed process displays relatively high chemoselectivity for C=O versus C=C hydrogenation as deduced from the retention of the latter in the conversion of olefinic ketones (Figure 5e) and aldehyde to the corresponding alcohols, however here in moderate yields (35–65%). Also of note is the limitation of redox side reactions, as hydrogen evolution, with good to very good Faradaic efficiencies (60–93%) obtained for the desired carbonyl reduction. By unlocking carbonyl hydrogenation at low overpotential, i.e., at a potential anodic or close to H^+^/H_2_ interconversion, the catalyst takes full benefit from the (modest) thermodynamic advantage of carbonyl hydrogenation over HER and thus limits (or avoids) undesired hydrogen production.

**Figure 6:**
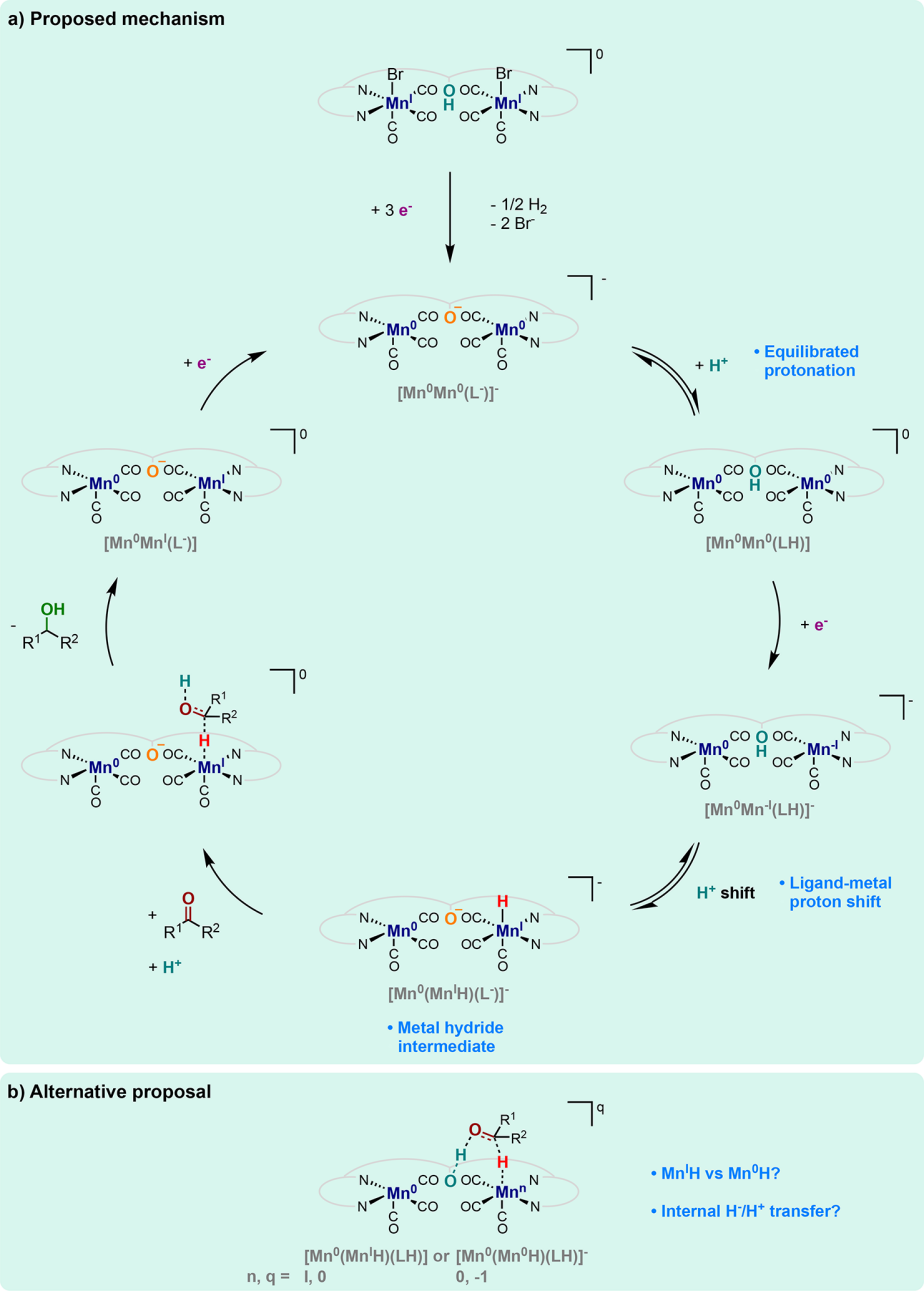
(a) Mechanistic proposal and (b) putative features in the hydrogenation of C=O bonds electrocatalyzed with [Mn_2_(**L**H)(CO)_6_Br_2_].

Mechanistic investigations were further pursued to bring insights into the origin of activity. By a combination of (spectro)electrochemical experiments and targeted variation of the catalyst structure, a mechanism has been proposed, which is summarized in Figure 6a. The proposed cycle turns over a central hydride intermediate **[Mn^0^(Mn^I^H)(L^–^)]^−^**. Favoring proton-coupled electron transfer (PCET) steps, the internal proton relay borne in the ligand phenoxy unit is key to reaching this hydride intermediate. In particular, protonation of the substituted phenolate in the entry intermediate **[Mn^0^Mn^0^(L^–^)]^–^**by external phenol is likely equilibrated, given that such two structurally similar units display proximal p*K*_a_ values.

This ligand-protonated **[Mn^0^Mn^0^(LH)]** intermediate in equilibrium can undergo a metal-centered reduction to the **[Mn^0^Mn^–I^(LH)]^−^** complex. Intramolecular proton exchange further affords the metal hydride complex **[Mn^0^(Mn^I^H)(L^–^)]^−^**.

The authors also hypothesize equilibration of this intramolecular proton shift based on the close acidity constants of molecular proxies of the Mn(I) hydride and the phenol unit.^60,61^ The importance of the internal proton relay at the phenol unit in electrocatalysis is underlined by the major loss of activity upon O-methylation at this position. The authors assume that the carbonyl substrate undergoes hydride attack from **[Mn^0^(Mn^I^H)(L^–^)]^−^** intermediate and intermolecular protonation, releasing the alcohol product and **[Mn^0^Mn^I^(L^–^)]**. The initial intermediate **[Mn^0^Mn^0^(L^–^)]**^–^ is then recovered after a one-electron reduction, closing the catalytic cycle. With this scenario, the authors favor protonation of the intermediate alkoxylate by an external proton donor (phenol).

In an alternative pathway, however, **[Mn^0^(Mn^I^H)(L^–^)]^−^** would undergo additional proton and possibly electron transfers providing the ligand-protonated **[Mn^0^(Mn^I^H)(LH)]** and **[Mn^0^(Mn^0^H)(LH)]**^–^ complexes (Figure 6b). Whether hydride transfer occurs from a Mn(I) or from a Mn(0) species is arguable in view of the literature precedent of thermal H_2_ hydrogenation catalysis with similar Mn complexes.^62,63^ However, such ligand-protonated intermediates display an ideal configuration for a concerted proton–hydride addition over the outer-sphere polarized C=O bond (Figure 6b).^64,65^ Interestingly, the binuclear nature of the complex is crucial for catalysis, as a mononuclear analogue reveals inactivity in electrochemical hydrogenation, but the role of the second Mn center remains an open question.

#### III.A.2 Coupling of Aryl Halides: Addressing the Redox State for C–C Bond Formation

**Figure 7:**
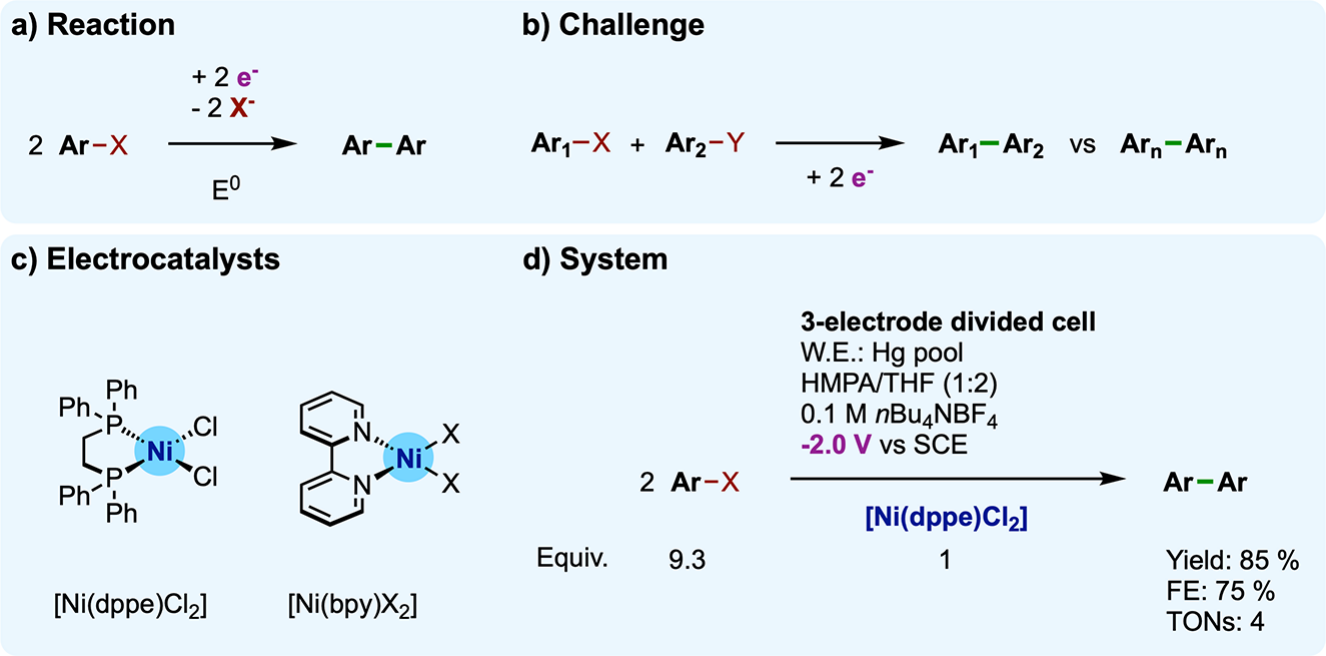
(a) Electrocatalytic Ullmann coupling of aryl halides: (a) reaction; (b) challenge; (c) molecular electrocatalysts [Ni^II^(dppe)Cl_2_] and [Ni^II^(bpy)X_2_] (d) used under conditions of mechanistic investigation (for [Ni^II^(dppe)Cl_2_]).

We address now a prototypical (electro)catalytic C–C coupling reaction, in which organometallic bonds are forged between a molecular catalyst and a substrate to generate intermediates that, in the right oxidation state, evolve the substrate. In particular, we discuss the case of nickel-catalyzed Ullmann homocoupling of aryl halides to biaryl compounds (Figure 7a,b).^66,67^

These couplings function at room temperature with Ni(II) halide catalyst precursors (typically [Ni(PPh_3_)_2_Cl_2_]) and stoichiometric amounts (versus the aryl halides) of chemical reducing agents, such as Zn metal powder. The reducing metal has double role in the reaction by (i) *in situ* generating catalytically competent low-valent Ni species from Ni(II) precatalysts and (ii) providing the stoichiometric amount of electrons required to perform the two-electron reduction of aryl halides into biphenyl (releasing two halides). The electrochemical investigation of Ni-catalyzed Ullmann coupling originally came in the late 1970s as an approach to elucidate the mechanism at stake.^68,69^

From a haloarylnickel(II) intermediate formed by oxidative addition (OA) of the aryl halide substrate on an *in situ* generated low-valent Ni(0) complex, the following activation of the second equivalent of substrate eventually yielding to biaryl formation was under debate.^70^ In particular, whether the OA of the second aryl halide would occur at a Ni(I) or a Ni(0) state and at the same or a distinct Ni center (monomolecular versus bimolecular pathway) remained open questions.^71^ To address these questions, Amatore and Jutand investigated the homocoupling of bromobenzene (PhBr) electrocatalyzed by the [Ni^II^(dppe)Cl_2_] complex (dppe = 1,2-bis(diphenylphosphino)ethane) as a model system (Figure 7c,d).^71−73^

**Figure 8:**
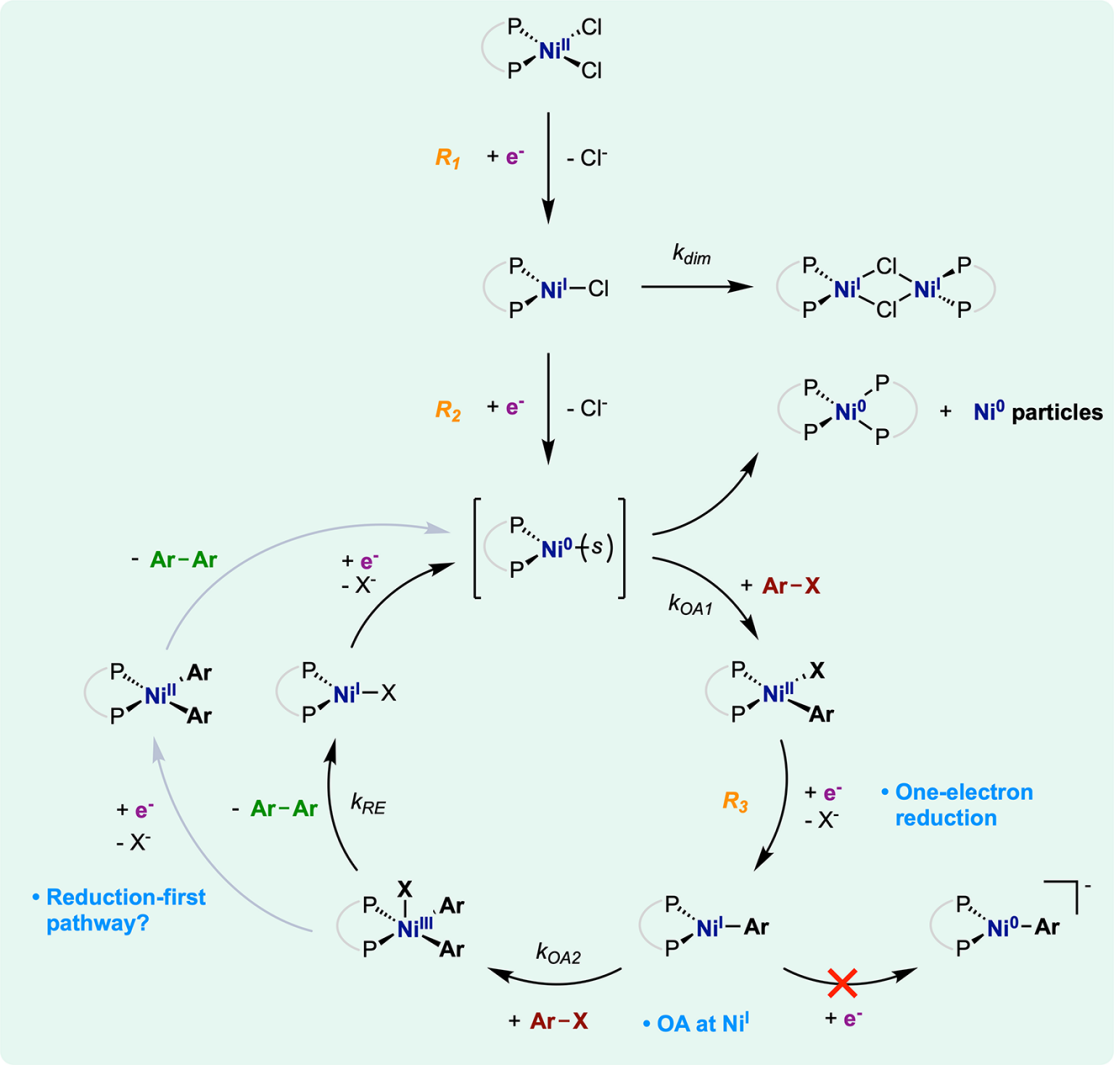
Mechanistic proposal for the Ullmann homocoupling of ArX electrocatalyzed by [Ni^II^(dppe)Cl_2_]. Gray arrows indicate the reduction-first route, as a possible alternative pathway. *s*, solvent.

The authors first explored the electroreduction of [Ni^II^(dppe)Cl_2_] using voltamperometry on a gold rotating disk electrode (RDE) or microelectrodes. In RDE voltamperometry, the pristine complex displays two reduction waves (*R_1_* and *R2*), with half-plateau potentials *E*_p/2_ = −0.77 respectively −1.36 V vs SCE. These waves correspond to two successive Ni-centered reductions associated with Cl^–^ loss, generating respectively [Ni^I^(dppe)Cl] and [Ni^0^(dppe)] transient intermediates (Figure 8). The unsaturated [Ni^I^(dppe)Cl] complex was found to undergo rapid dimerization (*k*_dim_ = 2.5 × 10^3^ M^–1^·s^–1^) into an electro-inactive Ni species (of the generic form [Ni_2_(dppe)_2_Cl_2_]). Yet, within short time scales (i.e., here at high scan rates) the reduction of [Ni^II^(dppe)Cl_2_] results in two consecutive, unperturbed reductions to [Ni^I^(dppe)Cl] and [Ni^0^(dppe)]. In addition, the expulsions of halides along reduction steps were found to be irreversible and fast. The generated low-valent, unsaturated [Ni^0^(dppe)] quickly disproportionates into a saturated [Ni^0^(dppe)_2_] complex and colloidal Ni(0) particles, but also constitutes a pertinent candidate to the OA of PhBr (Figure 8).

Indeed, the addition of 1 equiv of PhBr (vs [Ni^II^(dppe)Cl_2_]) triggers the appearance of a third one-electron-reduction event (*R_3_*) at a potential (*E*_p/2_ ≈ −1.8 V vs SCE) cathodic to the generation of Ni(0) complexes. This event was traced back to the reduction of a [Ni^II^(dppe)(Ph)Br] intermediate formed upon the OA of PhBr on the electrochemically generated [Ni^0^(dppe)]. Further investigation showed that this OA into [Ni^II^(dppe)(Ph)Br] is first order in [PhBr] and [Ni^0^(dppe)] and has a rate constant of *k*_OA1_ = 1.1 × 10^5^ M^–1^·s^–1^ (Figure 8).

Electrocatalytic conversion of PhBr is achieved by [Ni^II^(dppe)Cl_2_] only at potentials where [Ni^II^(dppe)(Ph)Br] is reduced, that is, negative to wave R_3_. For instance, electrolysis at −2 V vs SCE produces the diphenyl (Ph_2_) coupling product in 85% yield and 75% FE within ca. 6 h. By contrast, when electrolysis is performed at potentials cathodic to wave *R_2_* but anodic to wave *R_3_*, no coupling is observed but only a stoichiometric two-electron reduction of the starting Ni(II) complex. Hence, this Ni-catalyzed Ullmann coupling depends on the applied potential: formation of the [Ni^II^(dppe)(Ph)Br] intermediate (at *R_2_*) does not suffice for electrocatalytic turnover, which requires further reduction of this intermediate (at *R_3_*). In addition, the raise in electron consumption at wave *R_3_* with increasing PhBr excess further confirms that the electrocatalytic activity is linked to [Ni^II^(dppe)(Ph)Br] reduction.

The follow-up of [Ni^II^(dppe)(Ph)Br] in the electrocatalytic mechanism was then investigated. First, the electron count at *R_3_* is independent of the concentration in the initial Ni(II) complex, discarding electrocatalytic mechanisms that traverse a bimolecular rate-determining step (RDS) involving two Ni intermediates. At very fast scan rates (>100 V·s^–1^) outcompeting regeneration of the catalyst (*no catalysis* zone),^31^ the concentration in PhBr becomes limiting at wave *R_3_*, which saturates to one-electron stoichiometry. Thus, [Ni^II^(dppe)(Ph)Br] is reduced by one electron with concomitant Br^–^ expulsion to generate the [Ni^I^(dppe)(Ph)] complex as the next intermediate. In addition, further reduction of [Ni^I^(dppe)(Ph)] into [Ni^0^(dppe)(Ph)]^−^ is expected at a potential substantially lower than that of [Ni^II^(dppe)(Ph)Br] into [Ni^I^(dppe)(Ph)] (at *R_3_*), as the former implies electronic filling of an already occupied 3d-hybridized orbital in [Ni^I^(dppe)(Ph)] and the buildup of a negative charge on the complex. A catalytic scenario involving the [Ni^0^(dppe)(Ph)]^−^ intermediate is thus unlikely at *R_3_* where electrocatalysis develops and was also considered implausible by the authors (Figure 8).

The steps beyond the [Ni^I^(dppe)(Ph)] intermediate were investigated by employing conditions (excess PhBr and low scan rate RDE voltammetry) where the formation of this intermediate is non-rate-determining. Observations at low substrate concentrations reveal that the RDS is the second OA of PhBr on [Ni^I^(dppe)(Ph)], yielding a [Ni^III^(dppe)(Ph)_2_Br] intermediate. The authors found^72^ that the second PhBr equivalent adds at a rate (*k*_OA2_ = 960 M^–1^·s^–1^) expectedly slower than the first one. More importantly, this result discloses the involvement of a Ni(I)–Ni(III) route in the catalytic cycle. For comparison, the Pd counterpart electrocatalyst [Pd^II^(PPh_3_)_2_Cl_2_] instead requires a two-electron reduction of the first arylpalladium(II) intermediate into an anionic arylpalladium(0) species [Pd^0^(PPh_3_)_2_Ph]^−^ before the second OA proceeds.

At large PhBr concentrations, the second OA is no longer rate-determining. The electrocatalytic rate is now ruled by the intramolecular reductive elimination from [Ni^III^(dppe)(Ph)_2_Br] that forges the C–C bond of the diphenyl product and releases [Ni^I^(dppe)Br], at a rate of *k*_RE_ = 18 s^–1^ (Figure 8). The cycle closes by subsequent reduction of [Ni^I^(dppe)Br], largely favored at the potentials of electrocatalysis, that regenerates the [Ni^0^(dppe)] complex. Of note, in the case of electrocarboxylation of aryl halides, [Ni^I^(dppe)(Ph)] also constitutes the central intermediate from which CO_2_ addition takes place (electrocarboxylations across unsaturated C–C bonds are discussed in section III.C).

An alternative pathway involves the prior reduction of [Ni^III^(dppe)(Ph)_2_Br] into the Ni(II) 16-electron complex [Ni^II^(dppe)(Ph)_2_], which is likely feasible under the applied cathodic potential that is already sufficiently negative to reduce the initial Ni(II) 16-electron complex [Ni^II^(dppe)Cl_2_]. Then, a reductive elimination of Ph_2_ from [Ni^II^(dppe)(Ph)_2_] would yield [Ni^0^(dppe)] (Figure 8). This pathway was not discussed in the original work, but it may compete with the reductive-elimination-first one. The reduction-first route is likely minor, though, as the reductive elimination, which determines rate in excess substrate, is expected to be quite slower from the Ni(II) species than from the diphenyl Ni(III) intermediate.

Active electrocatalysts for this Ullmann coupling also extend beyond [Ni^II^(dppe)Cl_2_] to the [Ni^II^(bpy)X_2_] (X = Cl^–^, Br^–^, OMs^–^; Figure 7c) series, which mostly follows the same mechanism (in DMF),^74^ as reported by Devaud, Périchon, and co-workers.^74,75^ Of note, the electrocatalytic activity involving [Ni^I^(bpy)Ph] rises at lower overpotential (ca. 300 mV less negative) than that involving the [Ni^I^(dppe)Ph] counterpart, but at the expense of a slower turnover. This noticeable interplay between overpotential and activity will be further developed in sections III.B and IV.A.2. Besides, the [Ni^II^(bpy)X_2_] system broadens the approach to vinyl halide substrates^76^ and heterocoupling of substituted aryl products.^77^

#### III.A.3 Synthetic Opportunities through the Control of Potential

We highlight in this section that limiting the overpotential requirement with the help of molecular electrocatalysis can benefit the synthetic outcome. In particular, affording interconversion close to (just negative of) the standard potential of the targeted reduction discards side reactions having more negative standard potentials. This point is well exemplified in transformations involving the uptake of protons, for which hydrogen evolution is a common side reaction. For instance, the electrohydrogenation of many C–E (E = C, N, O) unsaturated bonds is expected at standard potentials more positive than proton reduction, as these bonds generally have exergonic hydrogenations. Thus, a potential window is accessible in which the former is thermodynamically favored whereas the latter is not. An electrocatalyst that achieves the desired hydrogenation with minor overpotential can thus allow operation in this window of potentials, discarding hydrogen evolution.

Furthermore, controlling the applied potential affords a fine-tuning of the redox states of the molecular electrocatalytic center, requisite to triggering the desired reactivity. This point is namely important in coupling reactions that involve OAs and for which accessing low-valent states at the metal center is crucial to activity. Simultaneously, controlling the driving force for reduction by setting a well-selected potential also limits over-reduction of the electrocatalyst, which may otherwise result in degradation of the catalyst or side reactions.

### III.B Improving Selectivity

#### III.B.1 C=C bond Formation in 1,2-Dehalogenation of Dibromoalkanes

**Figure 9:**
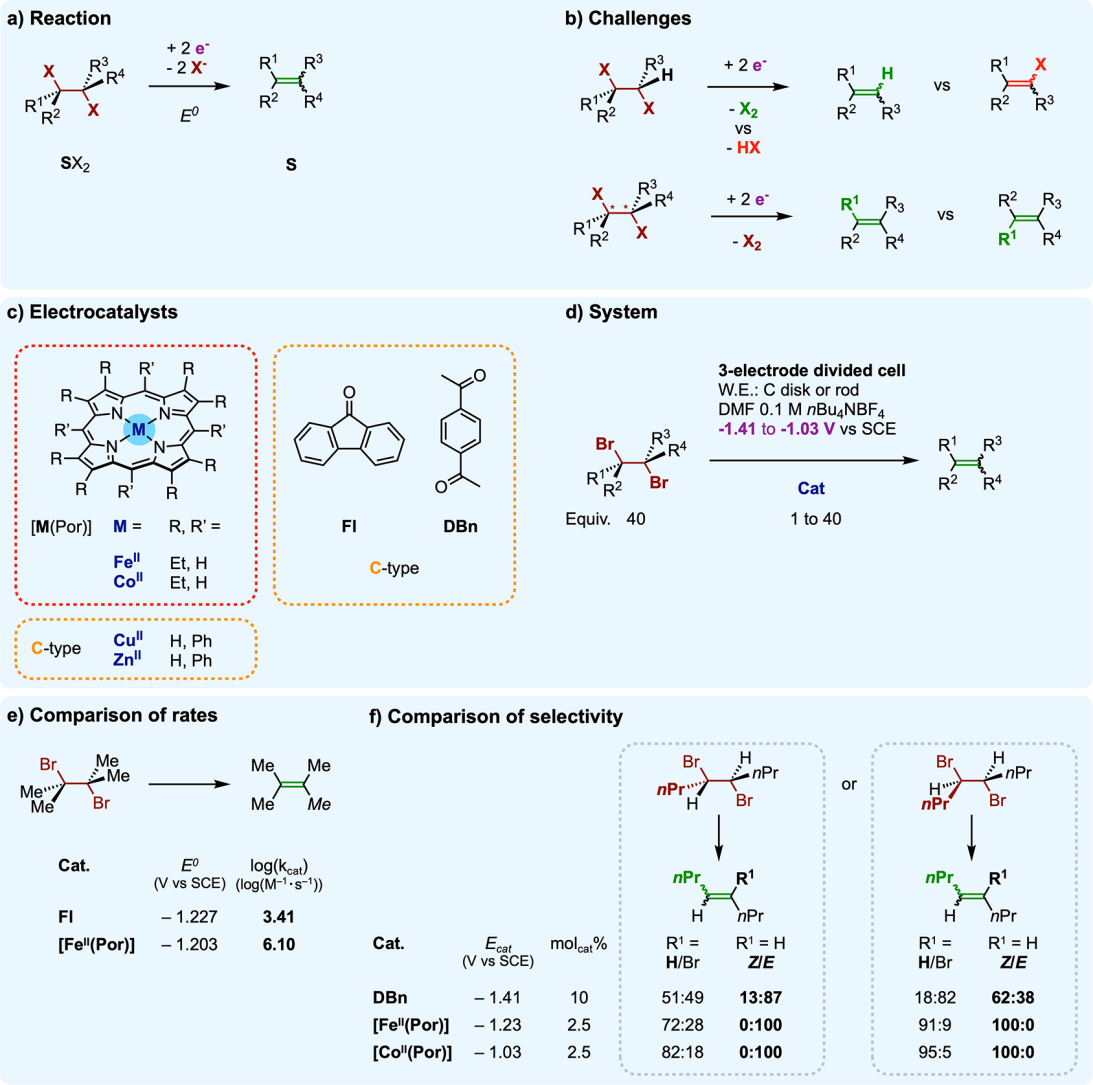
Electrocatalytic 1,2-dehalogenation of *vic*-dibromoalkanes: (a) reaction; (b) challenges; (c) molecular electrocatalysts (d) used under operating conditions with corresponding comparisons of (e) rates and (f) selectivity.

We now discuss a case that illustrates, beyond (over)potential questions, selectivity in electrocatalysis, viz., the reductive generation of olefinic C=C double bonds from parent vicinal dihalogenoalkanes X–C–C–X (Figure 9a,b).^78−82^ Whereas this transformation is often discussed with a focus on the cleavage of carbon–halogen bonds,^80,83^ we take in this Perspective the equally relevant view of the carbon–carbon π-bond formation. The electrochemical conversion of vicinal dibromoalkanes (**S**Br_2_) to olefins (**S**) has been studied by several groups from 1950 to today,^78−81^ but particularly thorough studies by Savéant and co-workers led to comprehending essential features that govern the electrocatalysis of this reaction.^79,84−86^ Despite having relatively positive standard potentials (typically within 0.1–0.2 V vs SCE), the direct two-electron reductions of vicinal dihalogenoalkanes at bare electrodes generally require very negative potentials to reach substantial catalytic conversion. For instance, overpotential requirements measured at the catalytic wave (at the average of peak and half-peak potentials) observed on a carbon electrode lay in the range 1.5–2.0 V for a series of aliphatic dibromoalkanes.^84^ This strong kinetic penalty is efficiently diminished by the introduction of molecular catalysts, with two principal classes being investigated. The first class comprises organic aromatics **C** (e.g., fluorenone **Fl** or diacetylbenzene **DBn**; Figure 9c), producing radical anions upon reduction, whereas the second class consists in 3d transition metal porphyrin type complexes (e.g., [**M**(Por)], **M** = Fe^II^, Co^II^; Figure 9c), undergoing metal-centered reductions.^84^ All these species display reversible reduction waves that evolve into electrocatalytic currents for olefin evolution in the presence of dibromoalkanes (Figure 9d).

However, plots of the rate constants as a function of the standard redox potentials of the catalysts, more conveniently derived into activation-driving force plots, evidence a clear gap between these two families. In the case of catalysts **C**, the data points comply with the MHL model of outer-sphere electron transfer. This matching classifies the **C** catalysts as redox catalysts. By contrast, [**M**^I^(Por)] complexes deliver activation-driving force relations away from the MHL model, which points to an inner-sphere (IS) operating mode ranking [**M**^I^(Por)] complexes as chemical catalysts (electrocatalysts).

We start by examining how the nature of the catalyst affects the performance for the electrogeneration of alkenes.

First, the electroreduction is from 10^3^ up to 10^6^ times faster with a [**M**^I^(Por)] chemical catalyst than with a **C** redox one operating at the same overpotential, as exemplified here with the electroreduction of 2,3-dibromo-2,3-dimethylbutane catalyzed by [Fe^II^(Por)] or fluorenone, **Fl** (Figure 9e). Conveniently synthesizing extrapolated data, the corresponding *catalytic Tafel plots*(18) provide an even clearer picture of this gap and definitely assess chemical catalysis by metal porphyrin complexes as intrinsically faster than redox catalysis with radical anions. This faster chemical catalysis applies over a large scope of linear, branched, and cyclic *vic*-dibromoalkanes, as precursors of terminal or internal *n*-substituted olefins (*n* = 0–4).

In addition, a major effect of the catalyst type on the selectivity of the reaction is also observed. We illustrate here on the prototypical electrogeneration of 4-octenes from *meso*- and (±)-4,5-dibromooctane stereoisomers using either redox (1,4-diacetylbenzene, **DBn**) or chemical ([Fe^II^(Por)] and [Co^II^(Por)]) catalysts. First, the chemoselectivity to 4-octenes is high with [**M**^II^(Por)] catalysts but heavily compromised with **DBn** because of monodebrominative side reactions evolving 4-bromo-4-octenes (Figure 9f). In addition, the conversion of *meso*- or (±)-4,5-dibromooctane diastereoisomers give full stereospecificity to respectively the (*E*)- or (*Z*)-olefin with [**M^II^**(Por)] catalysts, when **DBn** affords lower diastereoisomer excesses (Figure 9f). Inner-sphere catalysis thus unlocks chemoselectivity and stereospecificity here.

We now discuss the different mechanisms operated by the two classes of catalysts (chemical versus redox).

**Figure 10:**
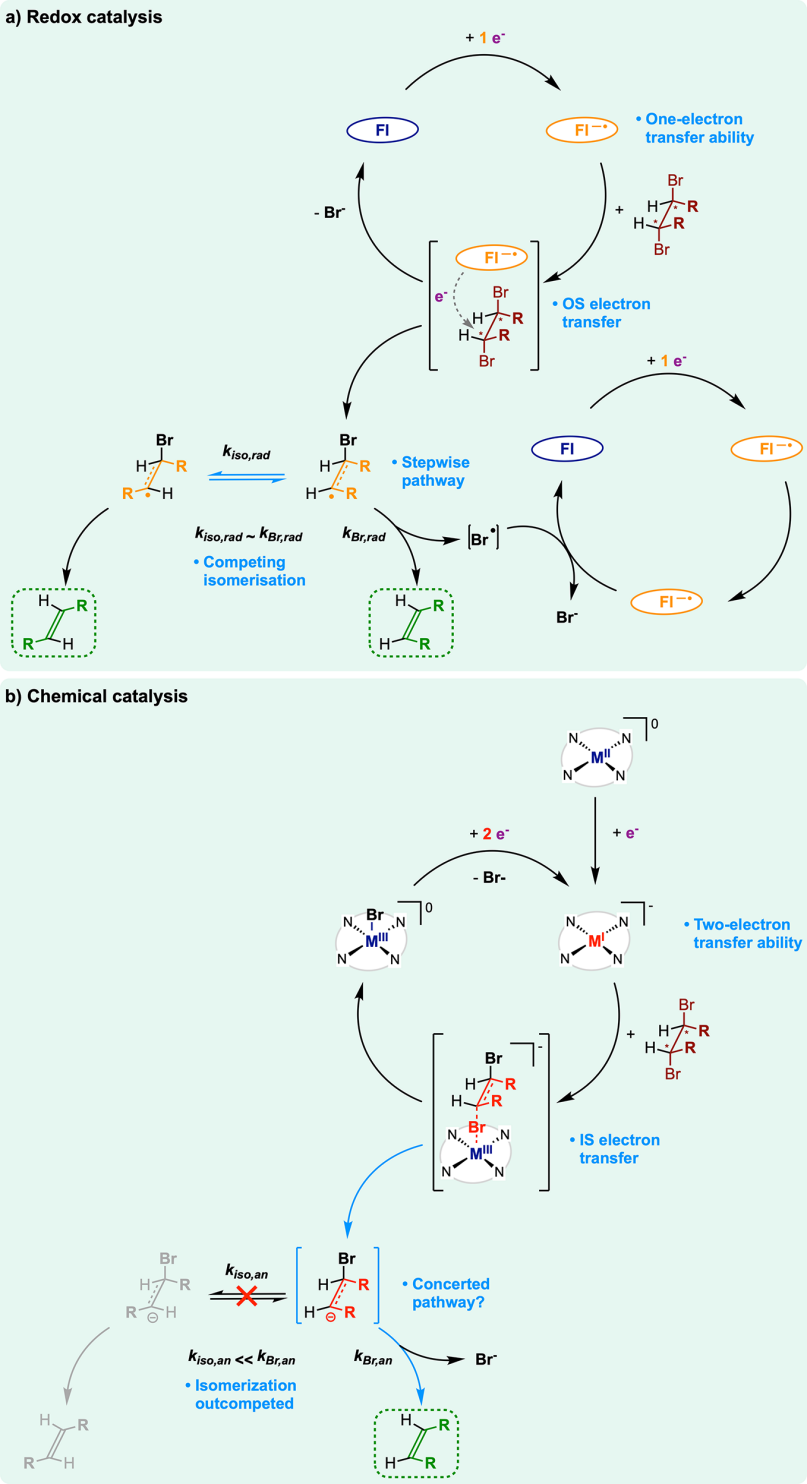
Mechanistic proposal for the 1,2-dehalogenation of *vic*-dibromoalkane with (a) redox catalysts and (b) chemical catalysts.

In the case of redox catalysts, the overall reaction occurs following two consecutive OS single electron transfers (Figure 10a). The first electron transfer is concerted with C–Br bond cleavage, releasing Br^–^ and leads to a β-bromoalkyl radical intermediate. The second reduction, by which the radical intermediate is converted into the final olefin, occurs at a potential substantially more positive (typically >1 V) than the first one and is proposed to be either concerted with Br^–^ expulsion or following stepwise the expulsion of Br^•^, at high, respectively low, catalyst loading. In view of the large driving force, this second step is substantially faster than the first dissociative electron transfer, which thus constitutes the rate-determining step. The OS character of these electron transfers subscribes to the MHL model and rules the kinetic penalty of the redox catalysts. Concerning stereoselectivity aspects, olefin evolution proceeds preferentially via antiperiplanar conformation of the starting *vic*-dibromoalkane. The *anti*-conformer is indeed the most easily reducible, since the generated radical intermediate is stabilized by an interaction between the p_*z*_ orbital hosting the unpaired electron and the σ* orbital of the vicinal C–Br bond. The rotation around the C–C bond of the radical (*k*_iso,rad_) is fast enough to compete with the second electron transfer (*k*_Br,rad_) (Figure 10a). This competitive isomerization process in turn generates partitioning between two isomers of the radical intermediate, with each of them yielding a distinct olefin diastereoisomer. Such a process explains why only partial stereospecificity is reached with redox catalysts.

We also note that redox catalysts investigated here are bound to a one-electron stoichiometry, although the reductive quenching of the **S**^•^Br radical intermediate upon transfer of a second electron from **C** is thermodynamically feasible (*E*^0^(**C**^+^/**C**^0^) are typically more negative than *E*^0^(**S**^•^Br/**S**;Br^–^)). Likely, the delocalization of this second electron over the catalyst π network raises strong kinetic penalties for the transfer to actually occur.

In the case of the chemical catalysis, the most favorable pathway goes via a halonium ion abstraction in an E_2_-type mechanism (Figure 10b). In this mechanism, the first and second electrons are delivered by the same metal center with sufficient reductive driving force. These two electrons are transferred in an IS fashion while Br^+^ is abstracted by the metal center, yielding a [**M**^III^(Por)Br] complex and the β-bromoalkyl anion intermediate. Expulsion of Br^–^ that generates the final olefin (*k*_Br,an_) occurs most likely in concert with halonium abstraction or is substantially faster than isomerization (*k*_iso,an_), thus rationalizing the full stereospecificity.

The large advantage of transition metal complexes acting as chemical catalysts over the redox ones can be traced back to at least two features of chemical reactivity. First, halonium transfer to the [**M**(Por)] catalysts has a high driving force, as a result of the strong affinity of the oxidized chemical catalysts (high-valent [**M**^III^(Por)]^+^) for halides, yielding a neutral [**M**^III^(Por)X] complex. This feature is absent with the redox catalysts, as oxidized radical anions do not possess a high affinity for X^–^. Second, reduced [**M**^I^(Por)]^−^ complexes concentrate the excess electronic density at the metal center (here in the d_*z*^2^_-based orbital). This localized excess of reducing equivalents enables delivering of the two electrons required for the generation of olefins in a very fast fashion, likely within the lifetime of a unique substrate–complex adduct, which warrants the stereospecificity.

As a final note, one could comment on the inner-sphere character of catalysis observed in the present case with chemical catalysts. The E_2_ mechanism implies that electron transfers occur in IS fashion within a [**M**^I^(Por)]^+^–**S**Br_2_ adduct at a transition state formed by M··Br··C bonding through a bridging bromide. However, this interaction has only mildly intimate character and remains isotropic regarding the M···Br bond (s orbitals), as sterically encumbered [**M**^II^(Por)] complexes provide similar rates. In particular, the coordination of the generated olefin to the oxidized [**M**^III^(Por)Br] metal center does not appear as a conceivable step in the mechanism. Also, whether or not the final expulsion of Br^–^ forming the olefin is concerted with the preceding electron transfers remains elusive.

#### III.B.2 Synthetic Opportunities by Addressing Catalytic Pathways

In this section, we have discussed how the interaction between the catalyst and the substrate (IS versus OS) is of major importance regarding the outcome of the synthetic transformation under study. First, chemical catalysts require lower overpotential to reach the same TOF than redox catalysts and thus provide better energy efficiency for the same electrocatalytic outcome. In addition, electrocatalysts can also drive the reductive synthesis through a more selective pathway, thus improving chemo-, regio-, or stereoselectivity. Such selectivity benefits from electron transfer(s) concerted with bond formation (or cleavage), a mechanism conceivably fostered by electrocatalysts operating inner sphere.

### III.C Building Up Molecular Complexity: Carboxylation of Unsaturated Carbon–Carbon Bonds

**Figure 11:**
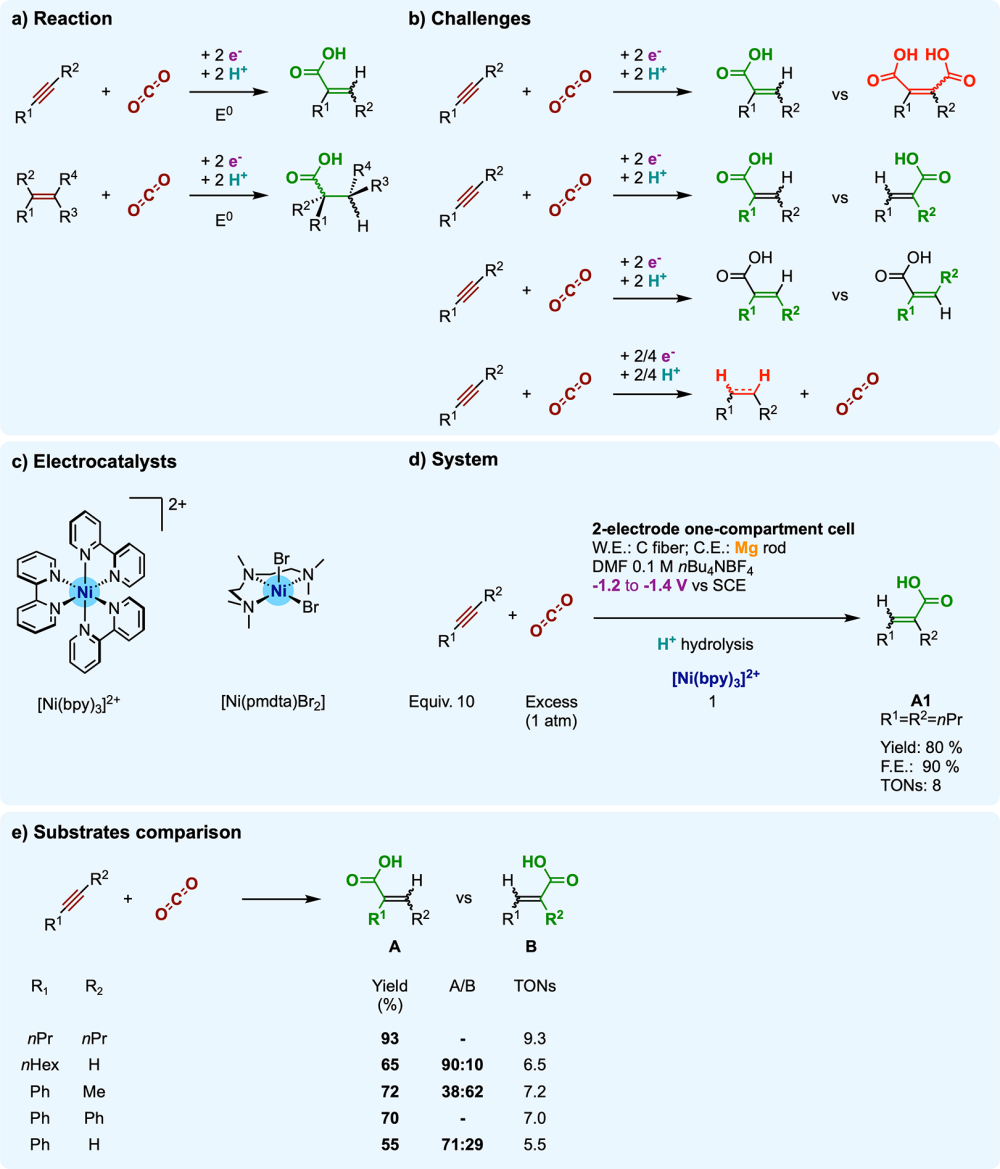
Electrocatalytic hydrocarboxylation of alkynes and alkenes: (a) reactions; (b) challenges; (c) molecular electrocatalysts [Ni^II^(bpy)_3_]^2+^ and [Ni^II^(pmdta)Br_2_] (d) used under operating conditions (e) to explore substrate scope (with [Ni^II^(bpy)_3_]^2+^).

In this last example, we discuss a case of IS electrocatalysis applied to the electrohydrocarboxylation (EHC) of unsaturated C–C bonds. This reaction consists in the electrochemical reductive coupling of alkenes or alkynes with CO_2_ to produce the corresponding hydrocarboxylated derivatives (Figure 11a).^3,87^ In terms of bond formation, electrohydrocarboxylation involves the reductive forging of a C–C bond and a C–H bond. In this multisubstrate reaction, chemoselectivity is a major challenge (Figure 11b). Indeed, both unsaturated C–C moieties and CO_2_ are prone to side reductions into, for instance, saturated C–C compounds and CO, respectively.

At the same time, ruling the number of carboxylation events is important to selectively access desired mono- or dicarboxylated products. In the common case of monohydrocarboxylation of unsymmetrically substituted C–C unsaturations, regioselectivity poses another challenge, as carboxylation can occur at both carbon positions.

#### III.C.1 Alkyne Electrohydrocarboxylation: An Early Example for the Approach

While the electrohydrocarboxylation of unsaturated C–C bonds has been the subject of a large number of works,^3,87^ a comprehensive and thoroughly studied electrocatalyzed version was disclosed in the late 1980s by Duñach and co-workers.^88−95^ The authors explored the EHC of various olefins and alkynes (including allenes,^88^ diynes,^93,94^ and enynes^95^) electrocatalyzed by molecular Ni complexes such as [Ni^II^(bpy)_3_](BF_4_)_2_ and [Ni^II^(pmdta)Br_2_] (pmdta = *N*,*N*,*N*′,*N*″,*N*″-pentamethyldiethylenetriamine) (Figure 11c–e). In particular, in-depth investigation of alkyne EHC using [Ni^II^(bpy)_3_](BF_4_)_2_ disclosed important findings regarding the selectivity, the role of cocatalysts, and the mechanism.^92^

**Figure 12:**
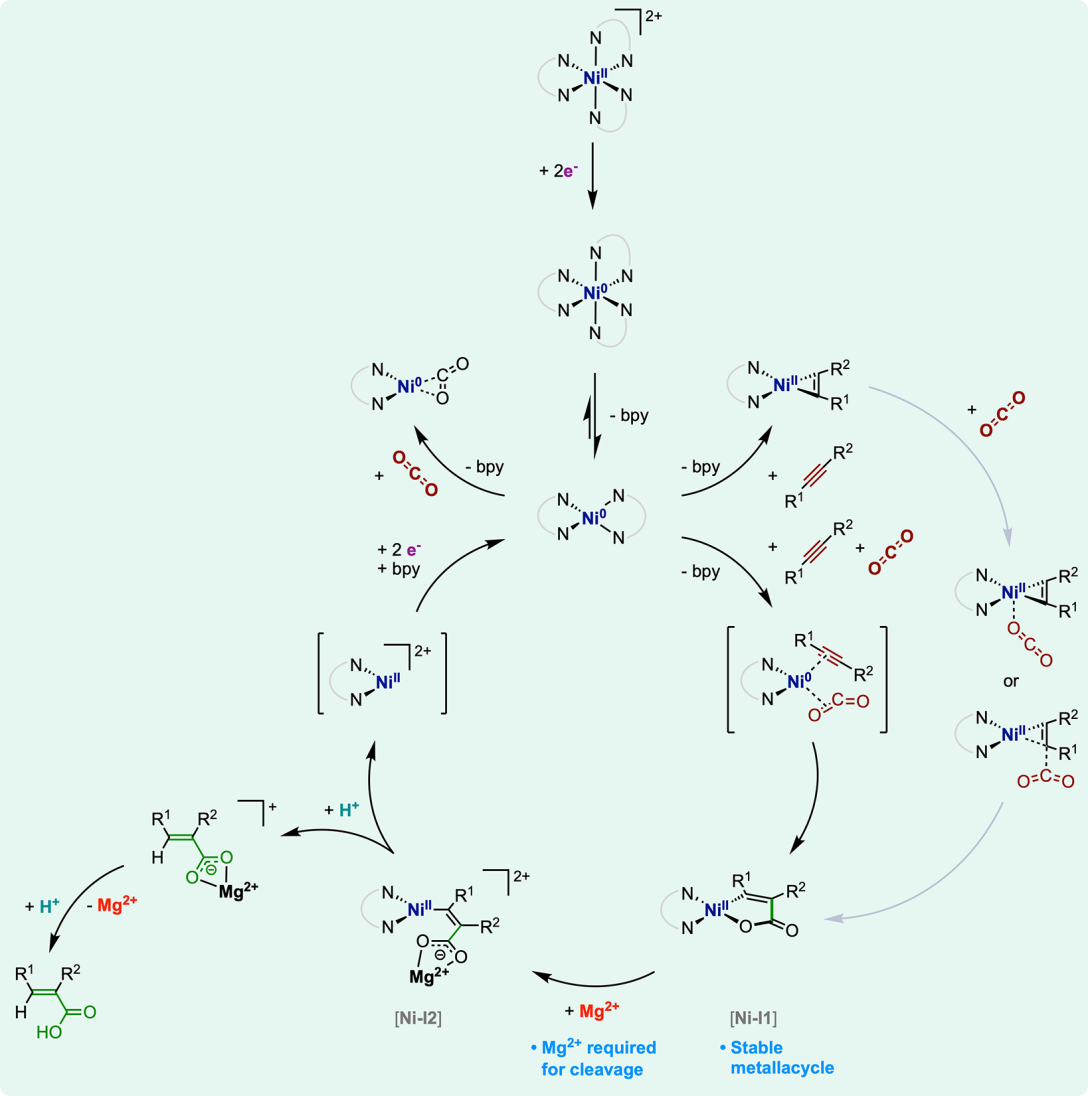
Mechanistic proposal for alkyne electrohydrocarboxylation (gray arrows indicate an alternative pathway).

Exploration of the electrochemical behavior of [Ni^II^(bpy)_3_](BF_4_)_2_ reveals that the pristine complex reduces in a partially reversible voltamperometric wave at −1.2 V vs SCE in DMF.^92^ The sequence underlying the electrochemical reduction of [Ni^II^(R_2_bpy)_3_]^2+^ (R = H for bpy or R = Me for Me_2_bpy = 4,4′-dimethyl-2,2′-bipyridine) is debated,^96,97^ but the generation of a two-electron-reduced transient complex [Ni^0^(R_2_bpy)_3_] is agreed upon. The electrocatalytically relevant pathways involve the subsequent expulsion of bipyridine (at moderate rate of ca. 10 s^–1^ for [Ni^II^(Me_2_bpy)_3_]^2+^)^97^ that produces a more stable tetracoordinated Ni(0) [Ni^0^(R_2_bpy)_2_] species (Figure 12).

In the presence of an alkyne (e.g., 4-octyne),^92,98^ electrochemical observations suggest that a fast ligand exchange follows the reduction to [Ni^0^(bpy)_2_] and affords a formal [Ni^0^(bpy)(alkyne)] complex (Figure 12). The structure of this intermediate is supported by isolated [Ni^0^(bpy)(alkyne)] samples,^99−101^ which display a square planar geometry and are best described as Ni(II) cyclopropene compounds. The electrochemical behavior of [Ni^II^(bpy)_3_]^2+^ under only CO_2_ indicates that the reduction to Ni(0) triggers the formation of a Ni–CO_2_ adduct,^92^ while electrocatalytic CO_2_ reduction occurs with [Ni^II^(bpy)_3_]^2+^ at potentials more negative^102^ than the ones explored here. The nature of the CO_2_ adduct is unknown, although an Aresta-like complex^103^ of the type [Ni^0^(bpy)(η^2^-CO_2_)] seems conceivable (Figure 12).

Observations in the presence of both CO_2_ and an alkyne (4-octyne)^92^ point to a concomitant binding of the two substrates at the Ni(0) state. Electrolyzing the alkyne and CO_2_ with catalytic amounts of [Ni^II^(bpy)_3_]^+^ at potentials of the Ni(II/0) wave (−1.2 to −1.4 V vs SCE), the authors noted that the outcome strongly depends on the experimental setup. In a two-compartment cell where the cathodic working electrode is separated from the anodic counter electrode (nickel wire) by a glass frit, the electrolysis consumes two electrons per Ni center and yields the EHC product (*E*)-2-propyl-2-hexenoic acid ((*E*)-**A1**; Figure 11d) in stoichiometric amounts versus Ni. By contrast, in a single-compartment cell fitting a sacrificial Mg anode (under otherwise identical conditions), (*E*)-**A1** is electrocatalytically evolved (and isolated under the form of the methyl ester derivative) in 10 TONs versus [Ni^II^(bpy)_3_]^2+^, with 80% yield versus 4-octyne and 90% FE (Figure 11d,e).

The possibility that Mg^2+^ cations released in solution by oxidation of the Mg anode promote electrocatalysis brought the authors to a mechanistic investigation. In a two-compartment cell, the stoichiometric reaction of [Ni^II^(bpy)_3_]^2+^ electroreduced at −1.2 V vs SCE with CO_2_ and 4-octyne generates a Ni species tentatively assigned to a 1-oxa-2-nickelacyclopentenone complex **[Ni-I1]** (Figure 12), by comparison with data of a synthetic sample. Such nickelacycle proves relatively stable and only opens under strong hydrolytic conditions into the hydrocarboxylation product (*E*)-**A1**. On the other hand, reacting a DMF solution of **[Ni-I1]** with anhydrous MgBr_2_ quickly cleaves the metallacycle and quantitatively affords the EHC product (*E*)-**A1** after mild hydrolysis. In addition, **[Ni-I1]** is an effective EHC electrocatalyst, but only in the presence of Mg^2+^ ions in the electrolysis mixture. These facts evidenced that Mg^2+^ ions are key for electrocatalysis by promoting the cleavage of the stable **[Ni-I1]** nickelacycle intermediate. The authors proposed that the metallacycle opens by a Ni^2+^/Mg^2+^ exchange at the Ni–O bond, generating a Ni–Mg vinyl-carboxylate bimetallic intermediate **[Ni-I2]** (Figure 12). From this bimetallic species, protonolysis of the Ni–vinyl bond can take place to generate Mg^2+^ carboxylate species, which accumulate as the primary EHC product.

The authors identified various H^+^ sources for protonation of the Ni(II)–vinyl bond: from residual water, from Hoffman degradation of the tetra-*n*-butylammonium cations (*n*-Bu_4_N^+^) of the supporting electrolyte, or from the DMF solvent itself. Of note, Brønsted acidity procured here by H^+^ is not sufficient to open the nickelacycle, which is only efficiently cleaved by Lewis acids (Mg^2+^ but also Zn^2+^ or Al^3+^). Finally, a two-electron reduction regenerates the Ni(0) intermediate that can engage in a new cycle. From these results, the catalytic cycle drawn in Figure 12 was proposed.

Whereas the general picture of the EHC mechanism is ascertained, several points remain subject to question. First, the (competitive) binding of CO_2_ and alkyne substrate on [Ni^0^(bpy)_2_] to form the nickelacycle **[Ni-I1]** is not quantified. We surmise a quick and highly favored formation of a Ni(II) cyclopropene intermediate, from which CO_2_ insertion can proceed to generate the nickelacyclopentenone. Whether CO_2_ would then insert in inner- or outer-sphere fashion is an open question, and the debate is notably ongoing in the related case of CO_2_ addition on Ni(I)–alkyl complexes.^104^ In addition, the absence of a magnified electrocatalytic current at the Ni(II/0) wave upon addition of both substrates indicates an overall slow turnover. Although the kinetics of the system is to date not quantified, the rate-determining step most likely lies in the opening of the nickelacycle, which can only be accelerated by Lewis acids. Finally, whereas a Ni^2+^/Mg^2+^ exchange at the Ni–O bond has been proposed, exchange at the Ni–C bond generating a vinyl Grignard-type intermediate prone to protonolysis seems a reasonable pathway, too.

The reach of this EHC approach was demonstrated on a range of internal and terminal alkynes, for which preparative electrolyses under mild conditions (RT to 80 °C; 1 bar of CO_2_) gave good to high yields for EHC products (Figure 11e). Several factors influence the selectivity metrics of the reaction toward EHC or within EHC products.

First is to be noted that here, as in the example discussed in section III.A.2, the potential applied during preparative electrolysis matters. The Faradaic efficiency to the 4-octyne EHC product is high when electrolysis is performed at the potential of the Ni(II/0) wave (−1.2 V vs SCE) but strongly degrades (by about half) when lower potentials are used (−1.7 or −2.2 V vs SCE), because of the competing CO_2_ electroreduction thriving under these more cathodic conditions. Also, alkyne (cyclo-)oligomerization is an important side reaction under EHC conditions, especially prominent with terminal alkynes, and likely catalyzed by Ni(0) intermediates, as reported in the literature.^99,105^ To circumvent that undesired reactivity, the authors operate under limiting alkyne concentration and slowly feed the substrate by a syringe pump over the course of the electrolysis.

The stereoselectivity is governed by the passage through a nickelacyclopentenone intermediate that forces *cis*-addition of CO_2_ and H^+^ across the C–C triple bond. As a result, the primary EHC products are (*E*)-olefins, but the outcoming selectivity is tamed due to isomerization catalyzed by Ni(0) species (*E*/*Z* 85:15 in 4-octyne EHC product).

For unsymmetrically substituted alkynes, two regioisomeric EHC products can be obtained. Terminal alkynes generally convert in α,α-disubstituted conjugated carboxylic acids as major products (typical α,α/α,β ratios between 70:30 and 90:10; Figure 11e). The invoked reason for the preferred α,α-disubstitution is the milder steric hindrance favoring the parent nickelacyclopentenone substituted in the β-position. With internal alkynes, the two regioisomers are obtained in mixtures close to equimolarity. In that case, the respective electronic influences of the substituents also strongly impart the formation of the metallacycle.

The substrate scope was further extended to conjugated and nonconjugated diynes^93,94^ and conjugated enynes^95^ using [Ni^II^(bpy)_3_](BF_4_)_2_ or (*in situ* formed) [Ni^II^(pmdta)Br_2_] as catalysts. Notably, mono-β-carboxylation of symmetrically 1,4-disubstituted 1,3-diynes is efficiently electrocatalyzed by [Ni^II^(pmdta)Br_2_] and produces the corresponding carboxylated *trans*-enynes ((*E*)-2-vinyliden-3-yne carboxylic acid) in good yields and selectivities.^94^ Instead, [Ni^II^(bpy)_3_](BF_4_)_2_ mostly affords the corresponding *cis*-1,3-enynes.

Despite the versatility of this EHC approach, some challenges are ahead. In particular, higher selectivities are still required for applications. Also, the oxidation of a sacrificial Mg anode to balance the reduction and provide the stoichiometric amount of Mg^2+^ ions required to evolve the carboxylate product imparts the resource and energy efficiency of the overall reaction. Bypassing the stoichiometric use of Lewis acid while pairing EHC with an electrocatalytic oxidation of interest would render the approach more virtuous.

#### III.C.2 Olefin Electrocarboxylation: Contrasting Catalyzed and Direct Electroreduction

**Figure 13:**
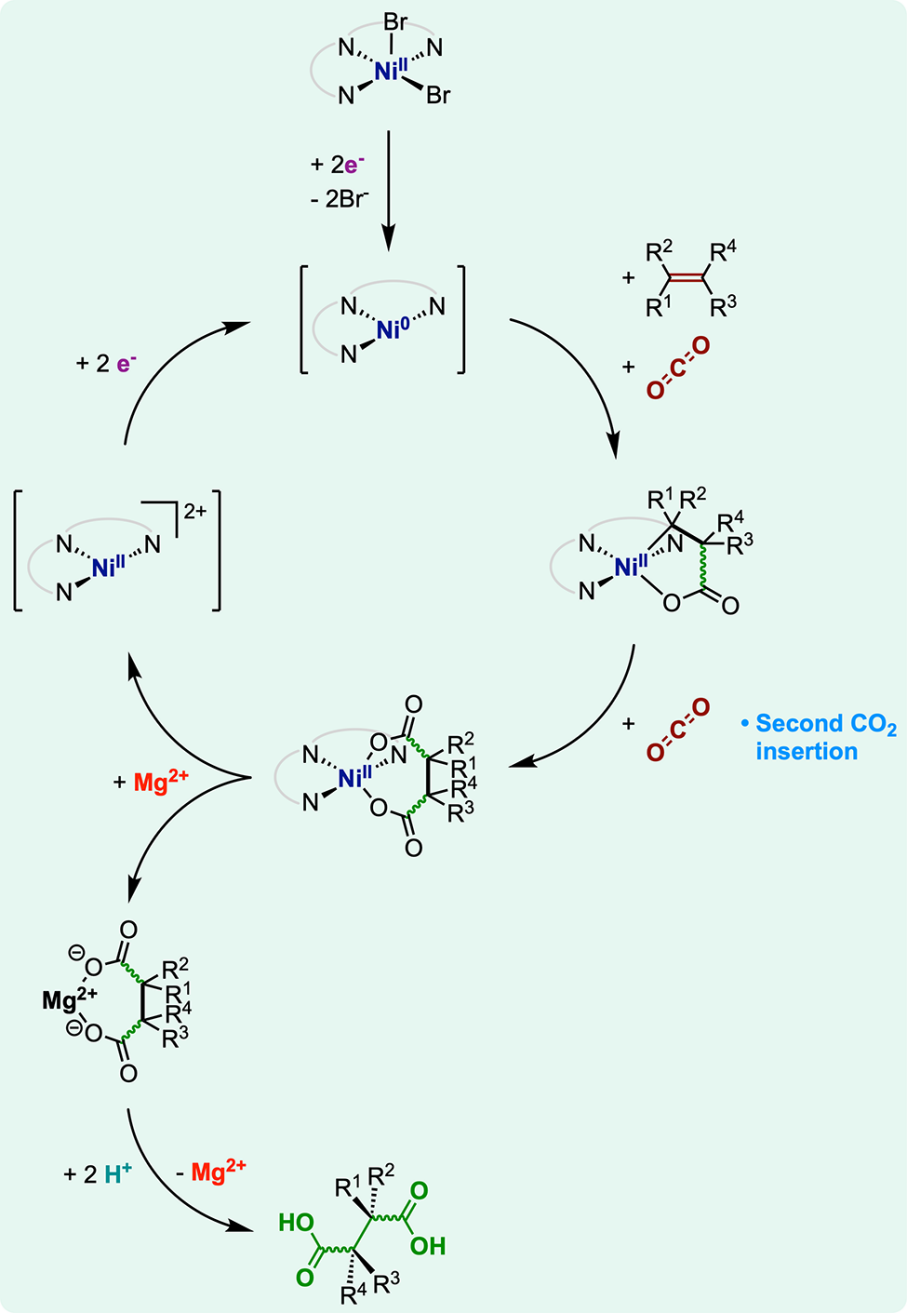
Mechanistic proposal for alkene electrocarboxylation catalyzed by [Ni^II^(pmdta)Br_2_].

The electro(hydro)carboxylation of olefins^106−109^ was also investigated. The reactivity is in general lower with the alkenes than with the corresponding alkynes, because of a weaker affinity of the C=C bond for the reduced Ni(0) complex.^101^ In that case, the *in situ* made [Ni^II^(pmdta)Br_2_] system (typically 10 mol %) was primarily investigated.^88^ While nonactivated aliphatic alkenes (e.g., ethylene, 1-octene, or 1,7-octadiene) proved unreactive, benzylic olefins (e.g., (α/β-methyl)styrene) could be transformed with moderate performance (typically 25–70% conversion and 50–80% FE). More interestingly yet, the conversion of alkenes predominantly produces the α,β-dicarboxylated saturated analogues, at variance with the alkyne substrates that generally forge the monocarboxylated EHC products. Such dicarboxylated products trace back to a twofold CO_2_ incorporation. The catalytic cycle likely proceeds via the buildup of a nickelacyclopentone intermediate (Figure 13); similar compounds are isolated elsewhere.^110,111^ The authors hypothesize that this nickelacycle is a candidate for a second CO_2_ insertion generating a 2,5-nickelacycloheptadilactone, from which Ni^2+^/Mg^2+^ exchange and protonolysis evolve the dicarboxylated products.

The origin of the twofold carboxylation has not been further investigated, but a lower affinity of alkenes for Ni(0) species may drag competitive binding in favor of CO_2_ and thus a higher degree of CO_2_ incorporation.

Of note, under these electrosynthetic conditions (carbon fiber cathode, Mg rod sacrificial anode, 10 mol % catalyst, constant 50 mA current) olefin conversion does not proceed without the Ni catalyst. This point is contrasted by recent works of Buckley and co-workers that report olefin electrocarboxylation in the absence of a catalyst.^112,113^ Their system relies on a single-compartment cell operated in a two-electrode configuration at 10 V applied voltage, using carbon-based electrodes and triethanolamine (TEOA) as a proton source.^112^ The approach selectively achieves the monocarboxylation of unsymmetrically substituted α-aryl alkenes, predominantly yielding β-carboxylated products. For instance, the prototypical styrene substrate is quantitatively converted into solely the corresponding monocarboxylic acids, with a high selectivity for β-carboxylation (1:8 α/β ratio). Not only terminal but also α,α- and α,β-disubstituted and trisubstituted internal α-aryl alkenes were transformed into mono-β-carboxylation products with high selectivity (typically 50–90% isolated yields with α/β ratios below 1:8). In these conditions, the authors propose a radical-based mechanism where the substrate (or CO_2_) is directly activated at the carbon electrode.

It is interesting that the Ni-catalyzed^88^ and direct reductions^112^ have distinct selectivities, with the former leading to dicarboxylated products and the latter leading to monocarboxylated ones. This point illustrates how the introduction of a molecular catalyst in the electrosynthetic conditions permits ruling the number of carboxylative events.

#### III.C.3 Opportunities in Electrohydrocarboxylation

Overall, the electrohydrocarboxylation of unsaturated C–C bonds gathers some of the most salient features of reductive electrosynthesis unlocked by transition metal complexes. This approach has high potentiality in terms of chemical scope, as carboxylic acids are ubiquitous intermediates or end compounds in commodity and fine chemicals. EHC also makes an advantageous use of CO_2_ by fixing that abundant, but overwhelming building block into added-value chemicals. In addition, chemical reductants engaged in classical hydrocarboxylations,^114,115^ such as molecular hydrogen at high pressure or sacrificial hydride donors (e.g., hydrosilanes or hydroboranes), are bypassed in the electrochemical version, thus affording a more direct, atom-economical access to carboxylic acids.

## IV Future Directions

**Figure 14:**
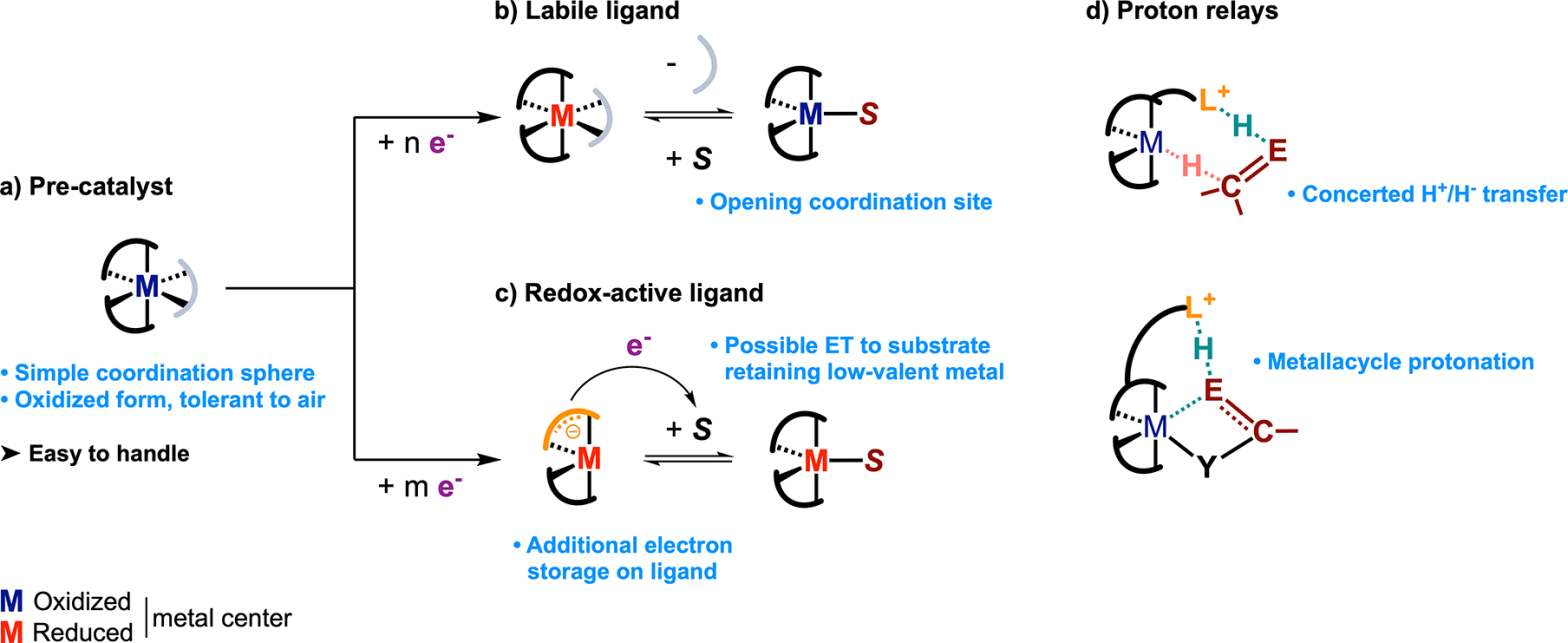
Design features for molecular electrocatalysts: (a) convenient precatalyst with ligands being (b) labile, (c) redox-active, or (d) integrating proton relays. ET, electron transfer.

Energy questions taken aside, a prominent possible advantage of molecular electrocatalysis for reductive organic synthesis resides in the mild operating conditions offered by the technique. In particular, most protocols discussed in the literature (as the ones exposed here) are performed at room temperature and ambient pressure. These protocols do not require the manipulation of either gases under high pressure or highly reactive reductants (hydrides, elemental alkalis). In addition, the preparation of highly reactive (pre)catalysts sensitive to the atmosphere is not required (Figure 14a). As such, the electrocatalytic approach gives access to a chemistry that is usually the apanage of low-valent, sensitive molecular catalysts (e.g., for C–C coupling) while discarding the tedious preparation and isolation of these catalysts. This latest point transpires in the structural simplicity of many electrocatalysts investigated to date for the reduction of organics, featuring simple ligands (commercial polypyridines, porphyrins, or chelating diphosphines) in conjunction with earth-abundant metal (Mn, Ni, Co, Fe) centers.

We now open our discussion to possible perspectives regarding molecular electrocatalysis in reductive organic synthesis. Taking lessons from the case studies developed above, we propose guidelines for the design of adequate molecular electrocatalysts before exposing synthetic opportunities provided by the approach.

### IV.A Designing Electrocatalysts

#### IV.A.1 Accessing the Active Site

Reductive electrosynthesis requires engaging at least two, and most often three, substrates (including protons) to forge bonds of interest (e.g., C–C, C–O) in added-value products (sections III.A.2. and III.C). These bonds forming at the metal center, for instance via reductive or β-hydride elimination, enough coordination sites should be accessible.

While (pre)catalysts displaying a readily open coordination sphere are challenging to handle, this difficulty is bypassed with the electroreductive dissociation of spectator ligands that leaves the catalyst in an activated, coordinatively unsaturated state (Figure 14b). This case is easily achieved from oxidized complexes bearing halides, as with [Ni(dppe)Cl_2_] in electrocatalytic Ullmann coupling (see section III.A.2), or strong σ-donor L ligands, such as bpy units in [Ni(bpy)_3_]^2+^ catalyzing EHC (see section III.C) or alkyne hydrogenation,^98^ that are expulsed when electron density at the metal increases.

The interplay of spectator ligands may actually reveal to be twofold. Indeed, activated complexes off the electrocatalytic cycle are potential catalysts for nonredox side reactions. Spectator ligands present (or in excess) in the electrosynthetic cell may quench these off-cycle activated complexes by closing the coordination sphere and inhibiting the side reactivity.

#### IV.A.2 Accumulating Electron Density

The next criterion in the design of relevant electrocatalysts is that several redox states can be readily and precisely addressed. Many redox electrosyntheses of interest involve a stoichiometry of at least two electrons *vis-à-vis* the substrate, which commonly—not always—leads to a catalyst spanning at least two oxidation states. This span is achievable within a moderate potential window for many transition metal complexes, in which the metal center can mediate these electron equivalents. The Ni-catalyzed aryl halide coupling (section III.A.2) clearly shows how the ability to dial in the desired redox state at the involved molecular catalyst is key to achieving the synthetic manifold.

Electroreductions involving more reducing equivalents or activation by a low-valent center would yet require highly reduced catalysts. In such cases, accumulating all the electronic density at the metal center is challenging and would possibly lead to side reactivity. Introducing redox-active ligands in the coordination sphere may reveal an appropriate strategy to act as an additional electron reservoir and reduce the electronic density centered at the metal^116−118^ (Figure 14c). This feature may in particular foster successive sequences of two-electron injections, as are often required for a selective reductive functionalization with simple building blocks (e.g., CO_2_, O_2_).

The interplay of redox-active ligands can also bring control of the type and sequence of electron transfers, thereby providing control of the reactivity. This point has been exemplified in a recent work by Toste, Chang, and co-workers that shows that the redox ability of ligands in Ni polypyridinic complexes tunes the electrocatalytic activity for the reduction of unsaturated alkyl iodides toward either cyclization or dimerization products.^119^ For the complex bearing a redox-active ligand, the reduced ligand induces an OS electron transfer to the substrate, which releases free iodide and an alkyl radical that can be trapped at the Ni center, favoring a controlled cyclization. By contrast, with the complex undergoing metal-centered reductions, an IS electron transfer to the substrate produces a nickel–iodide bond and ejects the alkyl radical, which is then prone to dimerization. It is interesting that an electron transfer chemistry primarily occurring from the redox-active ligand can preactivate the substrate while leaving the metal center mostly unaffected and readily available for downstream organometallic reactivity (Figure 14c).

However, the introduction of redox-active ligands always depends on the stability of the reduced ligand, which can undergo degradation by typically electroreductive hydrogenation or dimerization, and also shows a limit when excess delocalization on the ligand inhibits the metal-centered activity.^116,120^

A final but major point in the tuning of electron density is the underpinning of *scaling relationships* (also called *iron laws*)^42,121^ that link overpotential requirement and activity. Within a class of catalysts, more electron-rich reduced states lead to increased activity but at the expense of more negative reduction potentials. Breaking these relationships relies on the ingenuity of molecular design, and strategies developed in the frame of small molecule conversions have appeared in the literature^122^ (e.g., by introduction of proton relays,^123^ redox-active ligands,^124^ or electrostatic groups in the second coordination sphere^125,126^).

#### IV.A.3 Proton Relays

In addition, the electrocatalysis of redox reactions involving protons strongly benefits from the presence of proton relays in the coordination sphere, usually introduced under the form of protonatable amine, alkoxylate or thiolate groups. These moieties facilitate proton-coupled electron transfers (PCETs), which participate in lowering the overpotential requirement and increasing the activity, as notoriously exemplified for small molecule interconversions^127,128^ (e.g., H^+^, CO_2_, N_2_, O_2_). In addition to the promotion of PCETs, also important in electrosynthesis,^129^ proton relays can facilitate the electrophilic activation of a coordinated substrate. In a broad scope of electroreductive organic transformations involving the uptake of protons, for example, hydrogenation or hydroelementation, one can anticipate that proton relays would be a key structural feature to design suitable electrocatalysts (see section III.A.1).

For instance, proton relays may unlock the electrogeneration of ligand-protonated, transition metal hydride (H^+^L–MH^–^) species, allowing for a four-center concerted proton/hydride transfer, a central step in the hydrogenation of polarized C=E bonds^64^ (Figure 14d). But a fast protonation reached with well-tuned proton relays^127^ can also help the protonolysis of low-valent metal–alkyl or metallacycle intermediates formed in the course of electrocatalyzed reductions of unsaturated organics (Figure 14d).

### IV.B Synthetic Opportunities

We now discuss opportunities offered by molecular electrocatalysis from the viewpoint of reductive organic synthesis.

#### IV.B.1 Organometallic Electrocatalytic Perspectives in Organic Synthesis

**Figure 15:**
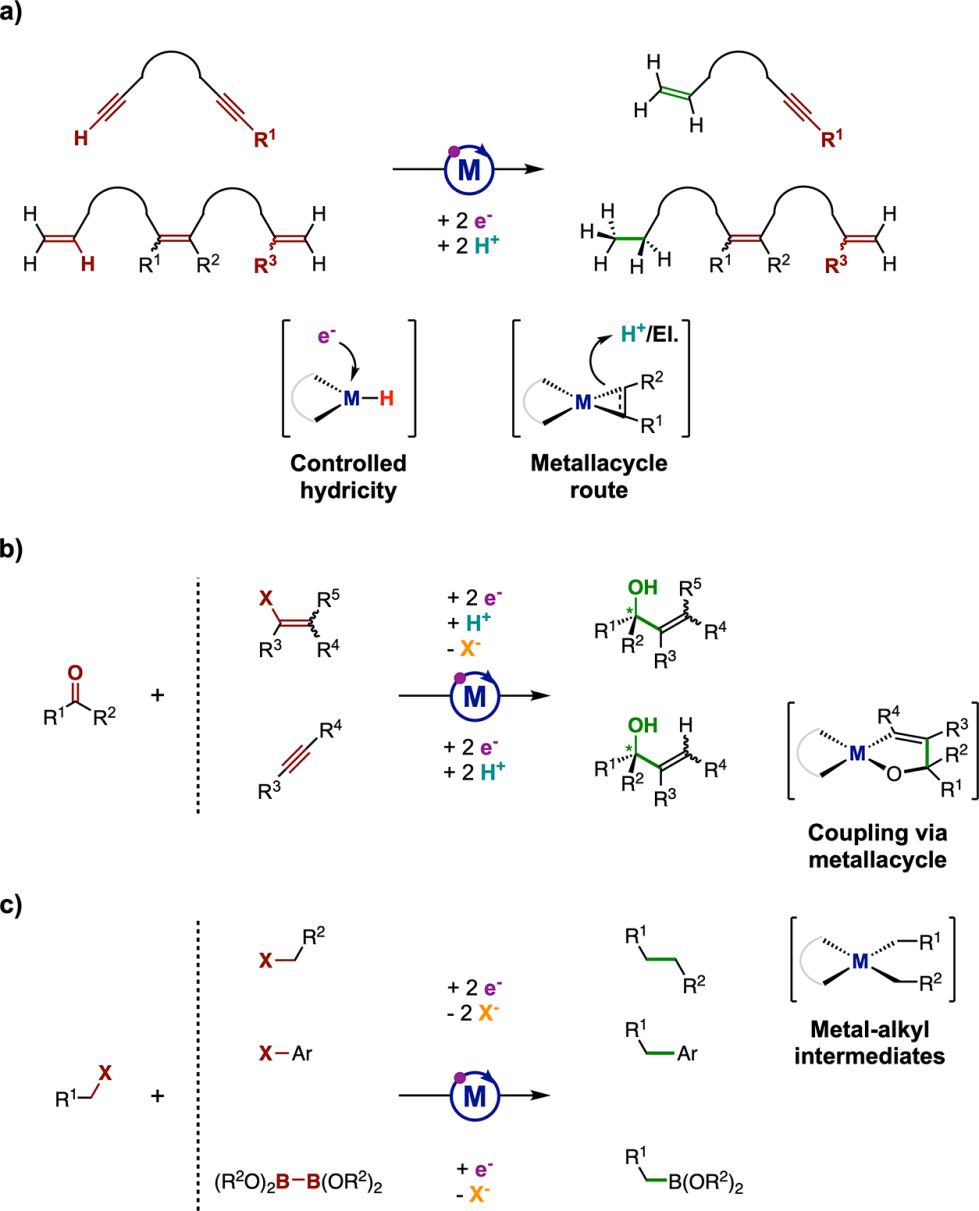
Opportunities for molecular electrocatalysis in (a) selective hydrogenation of polyunsaturated compounds, (b) ketone–vinyl halide and ketone–alkyne couplings, and (c) electrophilic carbon cross-couplings and borylation. El., electrophile.

First, the approach brings a new, original view of the hydrogenation chemistry of unsaturated organic bonds. For instance, the selective hydrogenation of organic substrates bearing multiple similar unsaturations (e.g., polyolefins or polyalkynes) is challenging due to the difficulty of finely controlling the chemical potential of the reducing agent/catalyst combination employed. This point is circumvented by application of an electrochemical potential and the use of protons,^130^ so as to obtain a reduced (hydride) complex whose redox state is precisely selected, and thus hydricity is well controlled (Figure 15a).

However, the possibility to separate delivery of electrons and protons in molecular electrocatalysis may also enable hydrogen transfer chemistry deviating from the classical H^–^ or H^•^ transfer schemes. First insights in that direction have recently been disclosed for the electrocatalytic selective semihydrogenation of alkynes with [Ni(bpy)_3_]^2+^.^98^ There, the hydrogenation cycle does not require the involvement of a hydride complex but can activate the alkyne at the reduced Ni(0) complex into a nickelacyclopropene intermediate that is further protonated. This metallacycle route opens up original activation ways toward hydrogenation and hydroelementation exempt of the need for hydrides (Figure 15a). But the electrochemical access to metallacycles would also open the portofolio of bond-forming reactions based on these intermediates, which are key steps in the synthesis of complexified organic backbones. This point was illustrated with the EHC of alkynes and olefins^88−95^ that build five- or seven-membered metallacycles starting from stable and accessible (Ni) complexes.

The field of C–C cross-coupling reactions, in which organometallic catalysts are integral, can also benefit from the mild conditions used in electrocatalysis that foster selectivity and group tolerance. While the electrocatalytic versions of many reductive cross-couplings have been known for a long time (see section III.A.2),^38^ the approach can extend to C–C forging from advanced synthons, as for instance between aldehydes and vinyl bromides (Nozaki–Hiyama–Kishi coupling)^131^ or ketones and olefins.^132^ Following this track while integrating some reminiscence of the EHC described above could for instance enable a molecularly electrocatalyzed coupling of ketones with vinyl halides or alkynes into tertiary vinyl alcohols (Figure 15b).

Another field where molecular electrocatalysis could prove particularly valuable is cross-electrophile C–C couplings, which bypass the synthetic steps otherwise required to generate carbon nucleophile derivatives. Electrosynthetic methodologies are currently explored toward the couplings between aliphatic and aliphatic or aromatic halides^133−137^ (Figure 15c), for some of which a precise control of the redox state of the molecular catalyst (by overcharge protection) was instrumental to increase selectivity toward the cross-products.^133^ Along these lines, electroreductive borylation^138^ through a molecularly catalyzed route would afford a functional-group-tolerant, mild installation of boronic esters from diversified alkyl halides (Figure 15c).

Finally, while some electrocatalytic reductions described here are reminiscent of established organometallic catalytic cycles and related intermediates, the possibility to precisely dial in redox states may give access to new intermediates, opening a landscape of unprecedented reactions. In this frame, a switch from the classical two-electron reactivity toward three- or four-electron reductions (the latter being already accessed with molecular electrocatalysts) is certainly a direction with potential for discovery.

#### IV.B.2 Functionalization with Building Blocks

An area where reductive molecular electrocatalysis can certainly have an important impact is the upgrading of organic backbones using abundant chemical resources. Particularly appealing are strategies to increase molecular complexity with simple, cheap, and readily available building blocks, in an atom-economical fashion.

**Figure 16:**
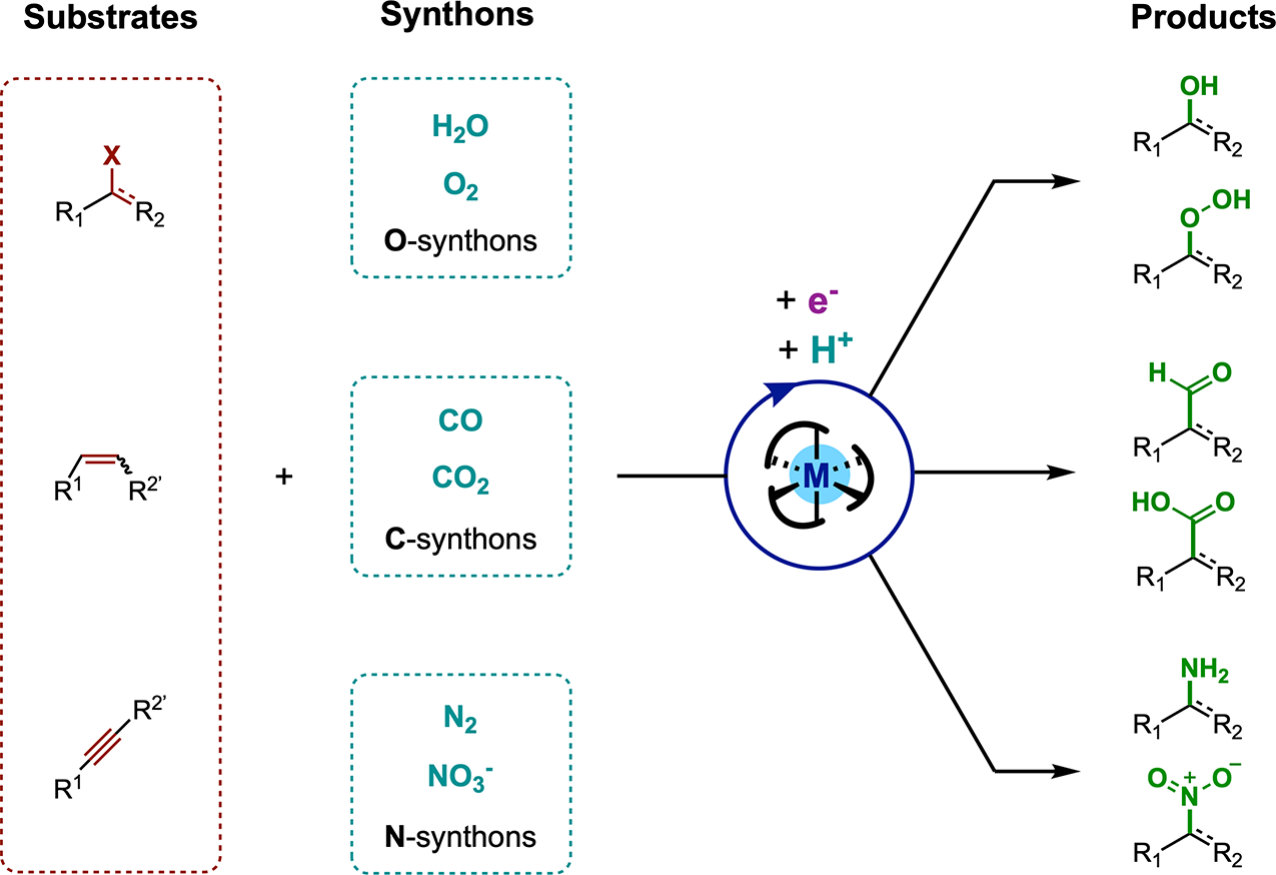
Building up molecular complexity from simple synthons.

These strategies involve for instance the electroreductive incorporations of functional groups deriving from one or the merging of several building blocks. The diversity of the parent resources give access to a handful of building blocks, for instance based on H, C, N, and O atoms leading to the electrocatalytic reductive forging of C–H, C–C, C–N, or C–O bonds (Figure 16).

The simplest of these resources is H_2_O, which also provides H^+^ required to balance many reductive reactions (e.g., in hydrogenation, hydrodimerization, hydrocyclization, hydroelementation). In the reductive frame, O_2_ is a direct source for oxygenated functions, such as alcohols or peroxides. For instance, the electrochemical activation of O_2_ into well-defined metal peroxo complexes was successfully applied for the oxo transfer to organic thiols.^139^ Inspiration from there can lead to electrocatalytic reductive peroxidation of organic backbones that avoids highly oxidizing conditions and thus fosters functional group compatibility. For instance, a molecularly catalyzed alkene electroperoxidation would bring an interesting alternative to the Isayama–Mukaiyama reaction,^140−142^ usually engaging silanes.

Concerning carbon-based groups, CO and CO_2_ provide convenient C_1_ bricks, namely to carbonyls or carboxylic acids. Namely, electrocarboxylative reactions discussed above can lead to highly valuable mono- or dicarboxylic acids, such as saturated dicarboxylic acids^143^ that are important monomers in the polymer industry. This molecular electrocatalytic construction of saturated dicarboxylic acids can be sourced from base chemicals, such as olefins, CO_2_, and protons, with a limited undesired hydrogenation. As a note, the implementation of asymmetric versions may also be accessed using chiral complexes.^144^

While derivatization with H^+^, O_2_, and CO_*n*_ can build upon the frame already established for the electrocatalytic reduction of the bare small molecules, perhaps more challenging is the incorporation of high-value-added N-based moieties (amine, imine, amides) from starting materials such as N_2_, NO_3_^–^, or N_2_O, with the latter being in addition interesting toward oxygenated functions. Furthermore, coupled functionalizations also open up a great deal of chemical creativity toward single-step multiple C–H, C–C, or C–E (E = heteroatoms) bond formations, as in the case of olefin electrohydrocarbocarboxylation.^145^ The parallel field of photoredox catalysis applied to synthesis has today very vivid developments,^7,146,147^ offering wide opportunities for cross-fertilization with the electrocatalytic method.

Finally, electrocatalytic reductive derivatization can advantageously extend to functionalities built on other elements (e.g., B, Si, P, S, halogens). While we centered our discussion on the transformation of carbon-based positions in organic skeletons, electrocatalyzed reductive conversions at other nonmetal or metalloid heteroatoms would also find interest. The reductive recycling of oxidized phosphines, boranes, or organoaluminum seems in this idea particularly appealing, for instance to regenerate the corresponding hydrides.

#### IV.B.3 Overall Reaction

Innately, the electrocatalytic approach has the advantage of supplying the redox equivalents (electrons or holes) from a renewable electrical resource. The use of abundant chemical building blocks discussed above makes steps toward chemical sustainability, which yet depends on the source of the other involved materials (organic starting materials, electrodes).

**Figure 17:**
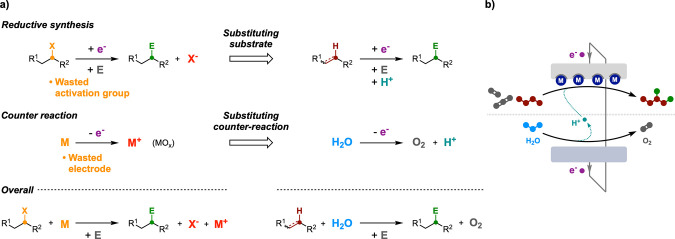
(a) Improving resource efficiency by substitution of substrates and counter reaction. (b) Molecularly engineered electrodes in flow cells.

The routes already explored to increase molecular complexity by electroreductive catalysis often rely on substrates bearing preactivated carbon positions. Activation of the targeted position, for instance with halogens, is aimed at facilitating conversion and controlling the regioselectivity of the reaction (on-site or remote). However, the prefunctionalization groups are eliminated in the course of the electrocatalytic process (for instance, as free halides X^–^ starting from C–X bonds), which jeopardizes the atom economy of the process. One possibility is to reutilize these moieties in the oxidative half-reaction of a paired electrolysis, as illustrated in the case of vicinal dehalogenation/halogenation.^82^ Another fruitful approach would achieve the desired reactivity through the electrocatalytic activation of compounds exempt of sacrificial prefunctionalizing groups (as is the case with alkyne EHC), to warrant high atom efficiency (Figure 17a).

In addition, the anodic half-reaction also matters in making the overall reaction sustainable. Many methodologies in electrocatalytic reduction of organics involve sacrificial anodes commonly made of bare metal such as Mg, Al, and Zn (see section III.C.1, for instance).^3^ These electrodes oxidize stoichiometrically into the corresponding M^*n*+^ ions or M_*n*_O_*m*_ oxides, resulting in a waste of valuable chemical resources. Carbon-based electrodes are also often implemented as auxiliary electrodes although they are prone to degrade (into CO or CO_2_) at high oxidative potentials. When materials more oxidation resistant, such as noble metals (e.g., Pt), are placed as anodes, oxidation of the solvent or supporting salt is seemingly taking place in a quantitative manner. To remedy this issue, productive substitutes can be found for balancing the electrocatalytic reductions (Figure 17a).

The simplest of all is likely water oxidation that consumes very abundant water and produces dioxygen while releasing protons that can be used in the reductive part. The oxygen evolution reaction (OER) can be performed at mild oxidative overpotentials with currently available catalysts and is namely used to balance fuel-forming electroreductive processes (hydrogen evolution, carbon dioxide reduction).^148−150^ Even more fruitful are paired electrolyses that balance the electrocatalytic reduction with an electrooxidation giving a product of interest.

#### IV.B.4 Device Implementation

Another challenge in the molecular electrocatalysis of reductive synthesis is in regard to the spatial distribution of reducing equivalents. Indeed, the electrocatalytically active entities are bound to a narrow reaction–diffusion layer in the vicinity of the electrode. Thus, only a fraction of the introduced molecular catalysts actually partake in electrocatalysis and activated catalysts escaping the reaction layer are prone to deactivation or undesired reactivity. The use of porous electrode materials (foam, paper, felt) giving a high electrode surface–bulk volume ratio offers a first opportunity to circumvent this issue. A step further would yet target the immobilization of the electrocatalyst onto the electrode surface, by means of anchoring. This strategy has been successfully applied to develop molecularly functionalized electrodes highly efficient for small molecule conversions.^151,152^ In that case, fast electron delivery is ensured for the surface-immobilized catalysts, which all participate in electrocatalytic turnover, whereas the diffusion of off-cycle catalysts in the bulk is avoided.

We note that the recent and intense development of single-atom electrocatalysts^153,154^ featuring molecularly defined centers also brings attractive candidates to the frame of reductive electrosynthesis. Functionalized and single-atom electrodes retain the molecular definition at the active sites, which is expected to warrant a high degree of selectivity in the electrosynthesis. In addition, the electrocatalytic entities stay bound to the electrode surface; these electrodes offer an ideal platform for implementation into electrochemical devices operated in flow, an important feature in the advent of catalyzed electrosynthetic processes (Figure 17b).

## V Conclusion

**Figure 18:**
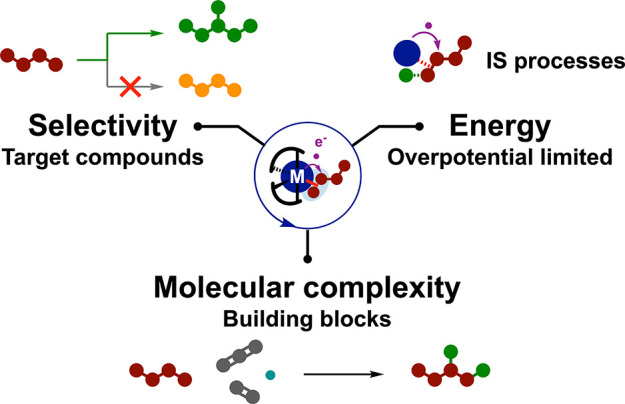
Opportunities brought by molecular electrocatalysis in electroreductive synthesis.

Electrocatalysis will take an increasing share in the synthesis of chemicals as electricity is rising up as a renewable energy vector. In this Perspective, we have showcased the reach of molecular complexes in electrocatalyzing the reductive transformation of organics (Figure 18).

Molecular catalysts are not only able to lower energetic costs but also address selectivity and reactivity by providing a large degree of control in reductive electrosynthetic conversions. Particularly instrumental are electrocatalysts that merge inner-sphere electron-mediating ability and organometallic catalysis. Facilitating both electrochemical and chemical steps along the electrocatalytic cycle, these features are key in original synthetic manifolds and in the upgrading of organic backbones with simple building blocks. The concepts highlighted here easily extend to a large body of redox reactions involved in the synthesis of base and fine chemicals (hydrogenation, elementation, couplings), for which thermal catalysis can serve as an inspiration point, but also to the conversion of biomass. This extension is today facilitated by the large scope of electrochemical techniques and theoretical analysis available for a fine evaluation and understanding of molecular electrocatalysts.

We hope that this Perspective motivates an interdisciplinary dialogue between molecular catalysis and electrochemistry to explore the potential for new synthetic methodologies harvesting renewable energy directly into the chemical value chain.
